# Altered plasma metabolite levels can be detected years before a glioma diagnosis

**DOI:** 10.1172/jci.insight.171225

**Published:** 2023-10-09

**Authors:** Sebastian Löding, Ulrika Andersson, Rudolf Kaaks, Matthias B. Schulze, Valeria Pala, Ilona Urbarova, Pilar Amiano, Sandra M. Colorado-Yohar, Marcela Guevara, Alicia K. Heath, Anastasia Chrysovalantou Chatziioannou, Mattias Johansson, Lars Nyberg, Henrik Antti, Benny Björkblom, Beatrice Melin

**Affiliations:** 1 Department of Chemistry,; 2 Biobank Reserach Unit, and; 3 Department of Radiation Sciences, Oncology, Umeå University, Umeå, Sweden.; 4 Division of Cancer Epidemiology, German Cancer Research Center (DKFZ), Heidelberg, Germany.; 5 Department of Molecular Epidemiology, German Institute of Human Nutrition Potsdam-Rehbruecke, Nuthetal, Germany.; 6 Institute of Nutritional Science, University of Potsdam, Nuthetal, Germany.; 7 Epidemiology and Prevention Unit, Fondazione IRCCS Istituto Nazionale dei Tumori, Milan, Italy.; 8 Department of Community Medicine, Faculty of Health Sciences, UiT The Arctic University of Norway, Tromsø, Norway.; 9 CIBER Epidemiología y Salud Pública (CIBERESP), Madrid, Spain.; 10 Ministry of Health of the Basque Government, Public Health Laboratory in Gipuzkoa, San Sebastián, Spain.; 11 Epidemiology of Chronic and Comunnicable Diseases Area, Biodonostia Health Research Institute, San Sebastián, Spain.; 12 Department of Epidemiology, Murcia Regional Health Council, IMIB, Murcia, Spain.; 13 Research Group on Demography and Health, National Faculty of Public Health, University of Antioquia, Medellín, Colombia.; 14 Institute of Public and Labor Health and Navarra, Pamplona, Spain.; 15 Navarra Institute for Health Research (IdiSNA), Pamplona, Spain.; 16 Department of Epidemiology and Biostatistics, School of Public Health, Imperial College London, London, United Kingdom.; 17 Nutrition and Metabolism Branch, International Agency for Research on Cancer (IARC), Lyon, France.; 18 Genomic Epidemiology Branch, IARC, Lyon, France.; 19 Department of Radiation Sciences, Diagnositc Radiology, and; 20 Department of Integrative Medical Biology (IMB), Umeå University, Umeå, Sweden.

**Keywords:** Metabolism, Oncology, Brain cancer

## Abstract

Genetic and metabolic changes in tissue and blood are reported to occur several years before glioma diagnosis. Since gliomas are currently detected late, a liquid biopsy for early detection could affect the quality of life and prognosis of patients. Here, we present a nested case-control study of 550 prediagnostic glioma cases and 550 healthy controls from the Northern Sweden Health and Disease study (NSHDS) and the European Prospective Investigation into Cancer and Nutrition (EPIC) study. We identified 93 significantly altered metabolites related to glioma development up to 8 years before diagnosis. Out of these metabolites, a panel of 20 selected metabolites showed strong disease correlation and a consistent progression pattern toward diagnosis in both the NSHDS and EPIC cohorts, and they separated future cases from controls independently of biological sex. The blood metabolite panel also successfully separated both lower-grade glioma and glioblastoma cases from controls, up to 8 years before diagnosis in patients within the NSHDS cohort and up to 2 years before diagnosis in EPIC. Pathway enrichment analysis detected metabolites related to the TCA cycle, Warburg effect, gluconeogenesis, and cysteine, pyruvate, and tyrosine metabolism as the most affected.

## Graphical abstract

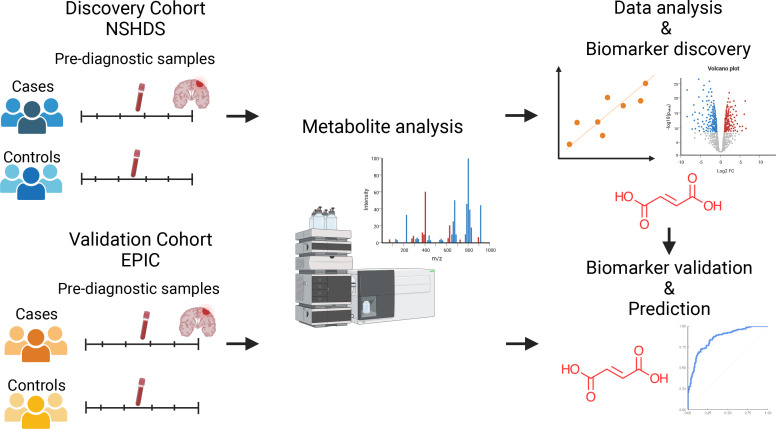

## Introduction

Gliomas, the most common type of malignant primary brain tumors, are usually detected late, when patients exhibit severe neurological symptoms such as seizures (1). Although treatment with surgical resection and concomitant radiochemotherapy have improved patient survival, prognosis for glioma patients is still poor. Patients suffering from the most common and most aggressive subtype — glioblastoma — have a median survival time of only 15 months (2). Since treatment options are limited, earlier detection of high-risk individuals could improve prognosis and affect patient survival (3).

Previous studies have shown that gliomagenesis starts several years before clinical symptoms appear (4, 5). Genetic aberrations causing glioblastoma tumorigenesis have been estimated to occur up to 7 years before diagnosis (4). Furthermore, a set of 15 metabolites in blood was associated with glioma progression up to 8 years before diagnosis (5), and a set of 9 metabolites were associated with higher glioblastoma risk even earlier (6). In both studies, the sets of metabolites indicated an imbalanced redox homeostasis (5, 6). In addition, it is well documented that elevated levels of the mitochondrial tricarboxylic acid (TCA) cycle metabolites fumarate, succinate, and D-2-hydroxyglutarate promote tumorigenesis (7).

In this study, we analyzed a large set of prediagnostic plasma samples from 2 independent cohorts, the Northern Sweden Health and Disease study (NSHDS) (8) and the European Prospective Investigation into Cancer and Nutrition (EPIC) study (9), from 18 study centers. The samples were collected 0.2–25 years before glioma diagnosis and were analyzed together with matched controls by global metabolomics analyses for discovery and validation of metabolic changes related to glioma development.

## Results

### Data overview.

**Figure 1:**
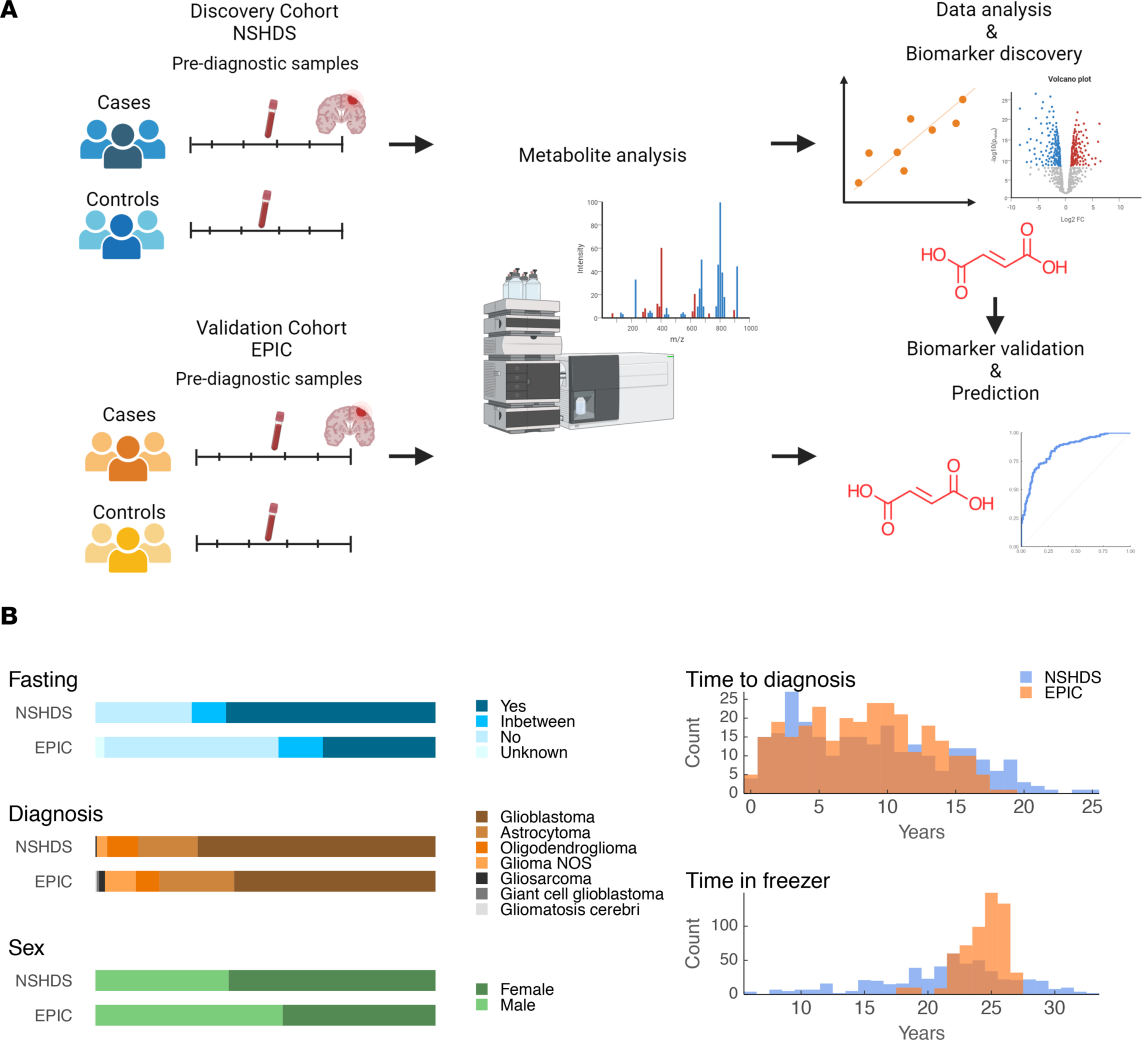
Study overview. (**A**) Overview of study design and workflow. Illustrations were created with BioRender.com. (**B**) Overview of cohort characteristics for NSHDS and EPIC samples.

**Figure 2:**
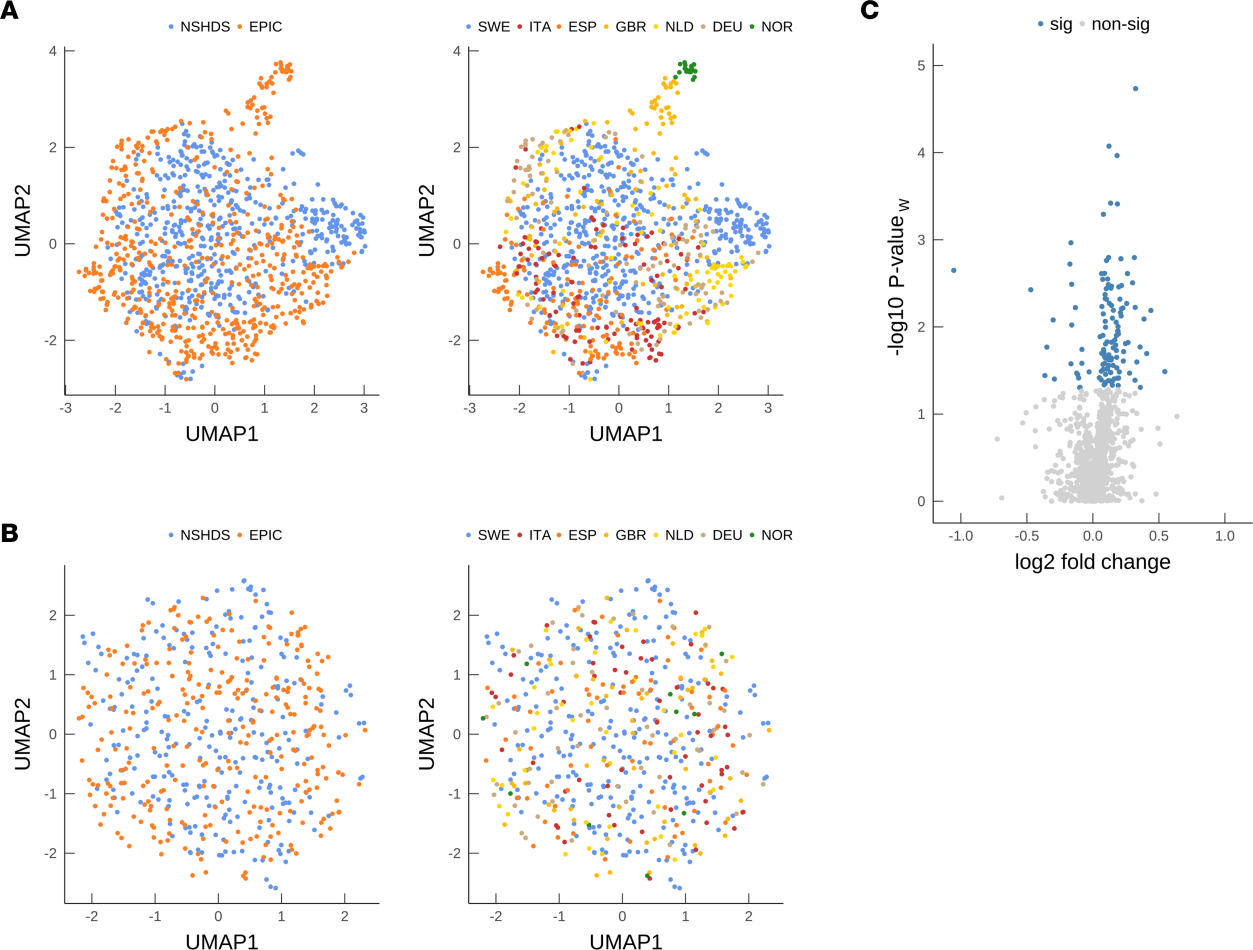
Data overview. (**A** and **B**) UMAP plots of plasma samples from NSHDS and EPIC. (**A**) Cases and controls (*n* = 1,100) colored by cohort (left) and sampling country (right). (**B**) Matched case-control pairs (*n* = 550) colored by cohort (left) and sampling country (right). SWE, Sweden; ITA, Italy; ESP, Spain; GBR, United Kingdom; NLD, Netherlands; DEU, Germany; NOR, Norway. (**C**) Volcano plot of detected molecular features in NSHDS within 8 years to diagnosis (*n* = 130 case-control pairs), with effect sizes and significance levels for each of the 1,061 molecular features as log-ratios. Significance was calculated by multivariate significance (2-sided, *P* value_w_ plotted) (28). Sig, Significant molecular features; nonsig, nonsignificant molecular features.

Study overview and description of study participants in the discovery (NSHDS) and validation (EPIC) cohorts are presented in Figure 1, A and B, and Tables 1 and 2. For case-control pairing, we employed stringent matching based on sex, BMI, age, time in freezer, fasting status, and study center. To obtain an overview of all 1,100 analyzed plasma samples from the global mass spectrometry–based (MS-based) metabolomics analyses, we first performed a Uniform Manifold Approximation and Projection (UMAP) for Dimension Reduction analysis (Figure 2, A and B). We included all metabolic features that were in common for both NSHDS and EPIC — in total, 802 metabolites (see Supplemental Methods for detailed information on data collection and curation; supplemental material available online with this article; https://doi.org/10.1172/jci.insight.171225DS1). UMAP plots of all samples, both future glioma cases (*n* = 550) and matched healthy controls (*n* = 550) from EPIC and NSHDS are shown as independent observations in Figure 2A and as dependent case-control pairs in Figure 2B. As anticipated, we observe cohort- and country-specific clusters when analyzing all cases and controls independently (Figure 2A), indicating systematic differences between and within the cohorts. This difference between cohorts and sampling countries was expected since samples were from multiple sampling centers with varying sampling routines and population differences. The study was therefore designed to reduce the impact of preanalytical differences by utilizing the differences in relative metabolite concentration between tightly matched case-control pairs. The UMAP plot constructed from an effect matrix of calculated metabolite concentration differences between matched case-control pairs (*n* = 550 pairs) shows that the overlap of samples from the cohorts greatly improves and that less cohort-specific clusters are observed (Figure 2B). This analysis shows the benefit of stringent matching of case-control pairs within the same cohorts, as a processing step before data analysis, to increase sensitivity for true biomarker detection and decrease both variation and false discoveries originating from preanalytical differences and covariates.

### Metabolites that indicate early glioma development.

**Figure 3:**
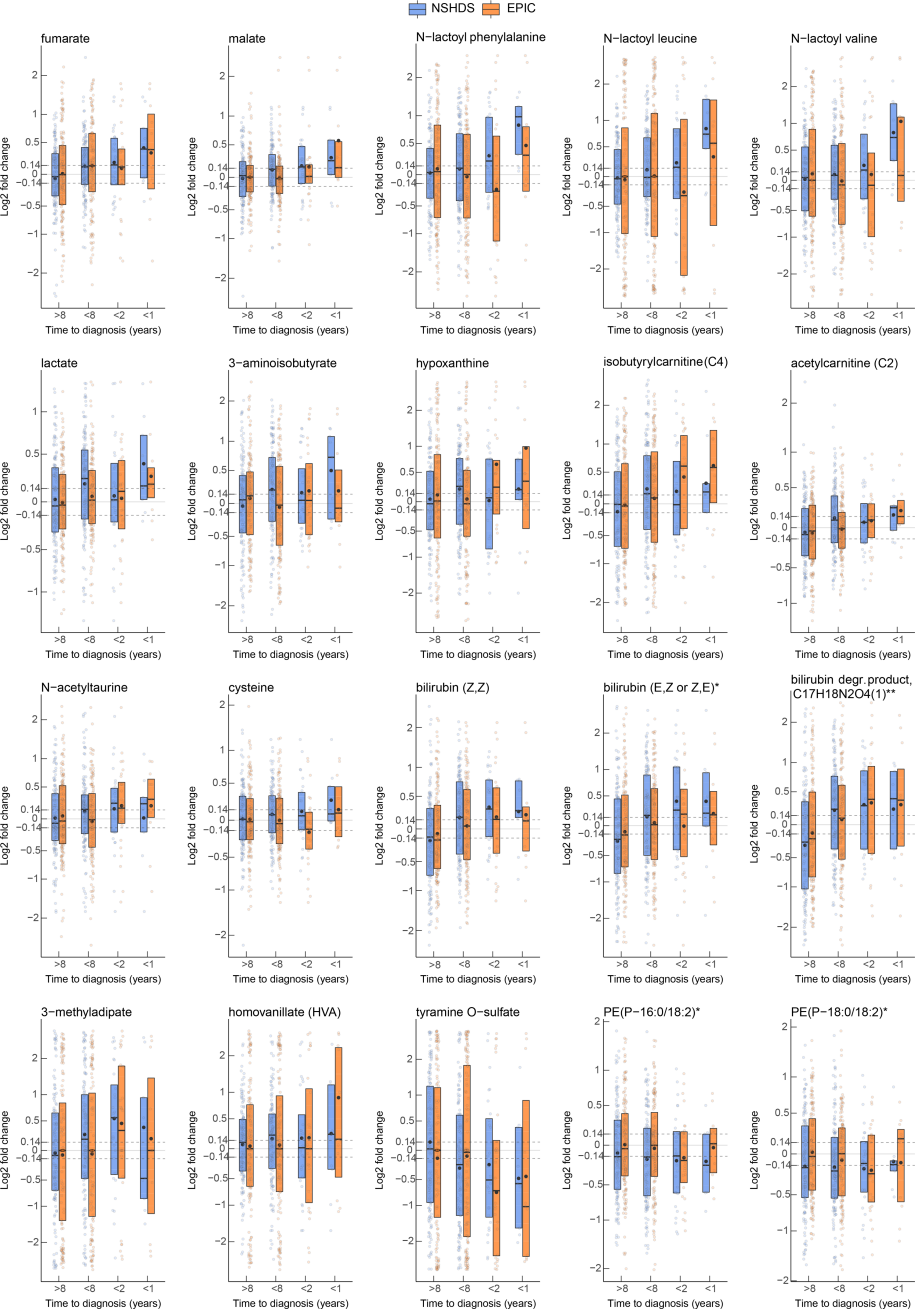
Metabolite levels for case-control pairs toward diagnosis. Box plots with average (dot) and median (line) fold change in case-control pairs for NSHDS (blue) and EPIC (orange) samples, subgrouped according to time to diagnosis (>8 years: NSHDS, *n* = 133, and EPIC, *n* = 148; <8 years: NSHDS, *n* = 130, and EPIC, *n* = 139; <2 years: NSHDS, *n* = 28, and EPIC, *n* = 28; <1 year: NSHDS, *n* = 9, and EPIC, *n* = 11). Dashed horizontal lines display a 10% difference. The *y* axis is nonlinearly transformed. All metabolite identifications were validated using synthetic standards, except putative identifications denoted * (confident identification without standard) or ** (putative identification without standard).

We used multivariate statistical analysis by means of Orthogonal Projections to Latent Structures – Effect Projection (OPLS-EP) to make use of the effect matrix obtained from matched case-control pairs and to discover metabolites related to glioma development. Since previous studies indicate that gliomagenesis starts up to 8 years before diagnosis (4, 5), we initially focused our analysis on case-control pairs sampled up to 8 years before diagnosis in NSHDS (*n* = 130 pairs). From the generated OPLS-EP model (CV-ANOVA; *P* = 0.005, R^2^Y = 0.46, Q^2^ = 0.08), 93 metabolites with known identity were found to reach statistical significance (Figure 2C and Supplemental Table 1). Of the 93 significantly altered metabolites (hereafter referred to as significant metabolies) found in NSHDS, 87 metabolites were also detected in samples from EPIC. However, in EPIC samples, only 1 of the 87 metabolites, fumarate, reached statistical significance (*P* = 0.02) when focusing on samples collected up to 8 years to diagnosis, while cystine was borderline significant (*P* = 0.06). It should be noted that plasma samples from EPIC were collected using sodium citrate as anticoagulant, which has been reported to induce matrix effects and quench metabolite signals (10, 11). Also, most of the EPIC samples were collected from nonfasting individuals, whereas most of the NSHDS samples were from fasting individuals (Figure 1B, Table 1, and Table 2), which could impact metabolite levels (12). However, our earlier study shows that the difference in levels of glioma-associated metabolites between cases and controls increases toward diagnosis (5). Therefore, we analyzed the metabolite levels toward diagnosis for the 87 of 93 significant metabolites that could be detected in EPIC, in order to examine if the glioma associated metabolites would be similarly altered closer to diagnosis. This analysis shows that 20 of 87 metabolites displayed the same direction toward diagnosis, with a mean difference of > 10% closer to diagnosis (within 2 years and/or 1 year to diagnosis), in NSHDS and EPIC (Figure 3 and Table 3). For most metabolites, the levels were higher in cases compared with controls, with the highest levels closest to diagnosis (Figure 3), except the levels of tyramine O-sulfate, PE (P-16:0/18:2) and PE (P-18:0/18:2), which were lower in cases. These had even more reduced levels closer to diagnosis (Figure 3), indicating a reversed molecular function. All significant metabolites for samples collected more than 8 years to diagnosis are listed in Supplemental Table 2.

To validate our findings of elevated lactate levels (Table 3 and Figure 3), we used the liquid chromatography–tandem MS–based (LC-MS/MS–based) Biocrates MxP500 quant platform for targeted identification and quantification of lactate levels in 354 NSHDS samples. Quantified μM levels of lactate were compared with the relative amounts from the Metabolon platform (Supplemental Figure 1, A and B). The methods showed strong correlation (*R*^2^ = 0.84), with elevated lactate levels in cases within 8 years to diagnosis and even higher levels closer to diagnosis (Supplemental Figure 1B). Lactate levels in samples that were not measured quantitatively were predicted using linear regression (Supplemental Figure 1C). The quantitative targeted measurements of lactate, including predicted levels, showed the same level of significance in case-control pairs within 8 years to diagnosis as seen for the untargeted measurement (*P*_untargeted_ = 0.0004, *P*_targeted_ = 0.0004).

### Predicting glioma development.

**Figure 4:**
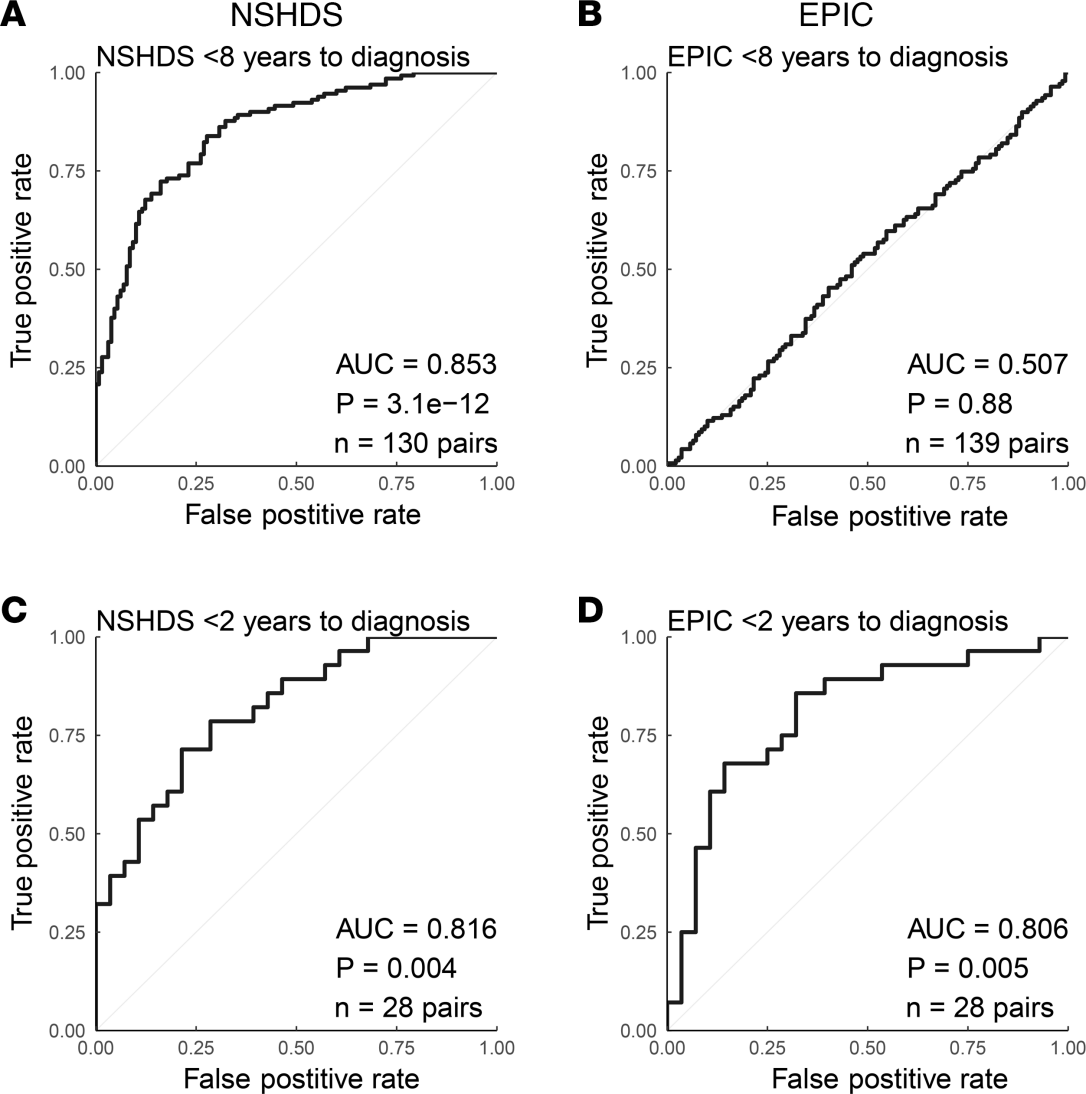
ROC analysis using a panel of 20 metabolites. (**A**–**D**) Glioma case-control pairs sampled less than 8 years before diagnosis in NSHDS (**A**) and EPIC (**B**), and less than 2 years before diagnosis in NSHDS (**C**) and EPIC (**D**). Wilcoxon signed-rank test (2-sided) was used to calculate the significance of the ROC curves. *n* = number of pairs available for each analysis.

To assess if the panel of 20 selected metabolites with consistent progression pattern toward diagnosis in both NSHDS and EPIC could predict glioma development, we first generated an OPLS-EP model using the metabolites and the 130 case-control pairs in NSHDS sampled within 8 years to diagnosis. The predictive ability of the model was assessed by predicting the samples from NSHDS, used to generate the model, and samples from EPIC that were not used to generate the model. The results were evaluated with ROC analyses (Figure 4). Within 8 years to diagnosis, the panel of 20 metabolites predicted case-control pairs in NSHDS well, with an AUC of 0.853 and *P* = 3.1 × 10^–12^ (Figure 4A) whereas case-control pairs in EPIC showed a poor prediction with an AUC of 0.507 and *P* = 0.88 (Figure 4B). However, prediction limited to case-control pairs within 2 years to diagnosis in EPIC displayed an AUC of 0.806 with a significant *P* value of 0.005 (Figure 4D). Similar results were observed for case-control pairs within 2 years to diagnosis in NSHDS (AUC = 0.816, *P* = 0.004) (Figure 4C).

**Figure 5:**
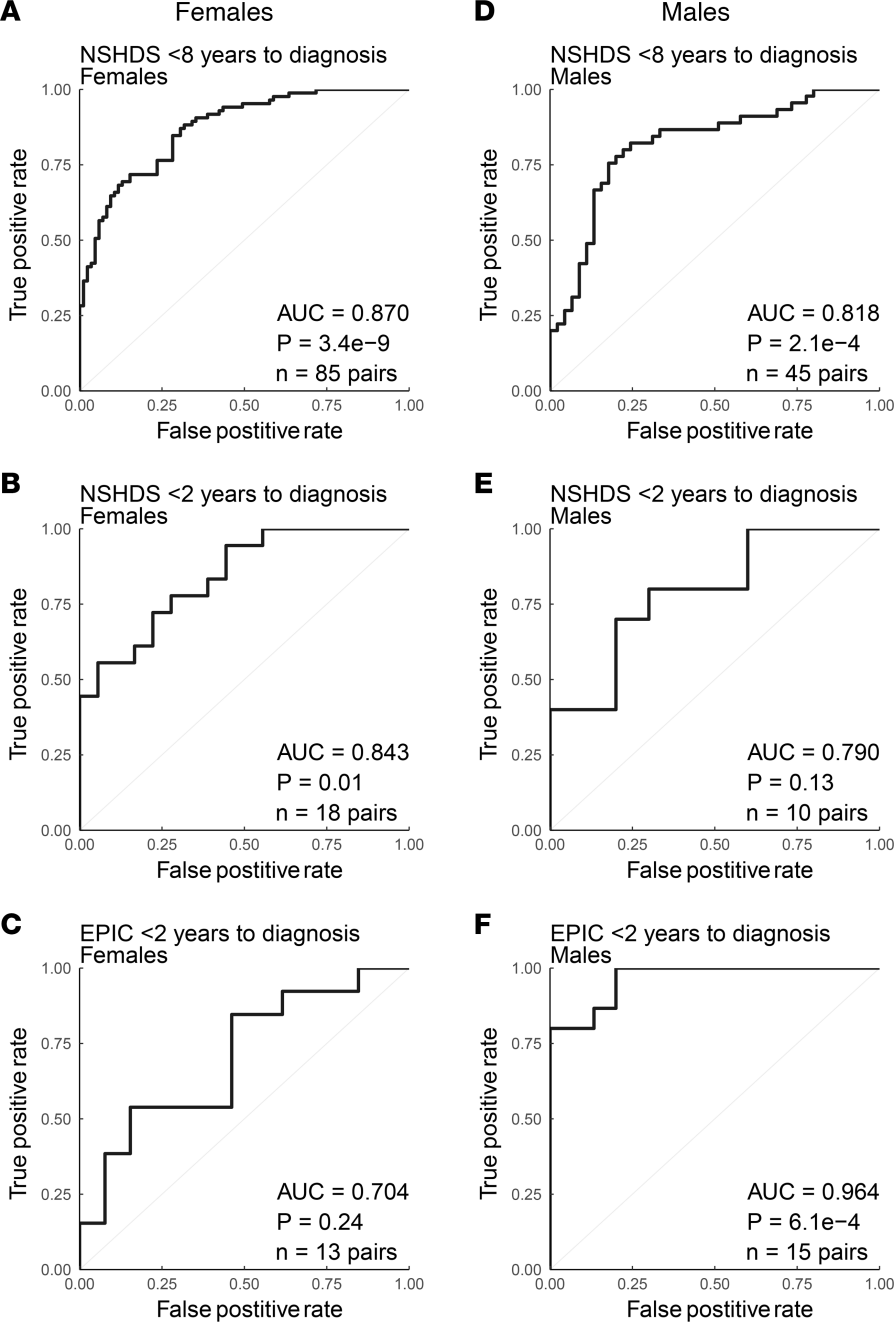
ROC analysis using a panel of 20 metabolites for females and males. (**A** and **B**) NSHDS female case-control pairs sampled less than 8 years (**A**) or less than 2 years (**B**) before diagnosis. (**C**) EPIC female case-control pairs sampled less than 2 years before diagnosis. (**D** and **E**) NSHDS male case-control pairs sampled less than 8 years (**D**) or less than 2 years (**E**) before diagnosis. (**F**) EPIC male case-control pairs sampled less than 2 years before diagnosis. Wilcoxon signed-rank test (2-sided) was used to calculate the significance of the ROC curves. *n* = number of pairs available for each analysis.

**Figure 6:**
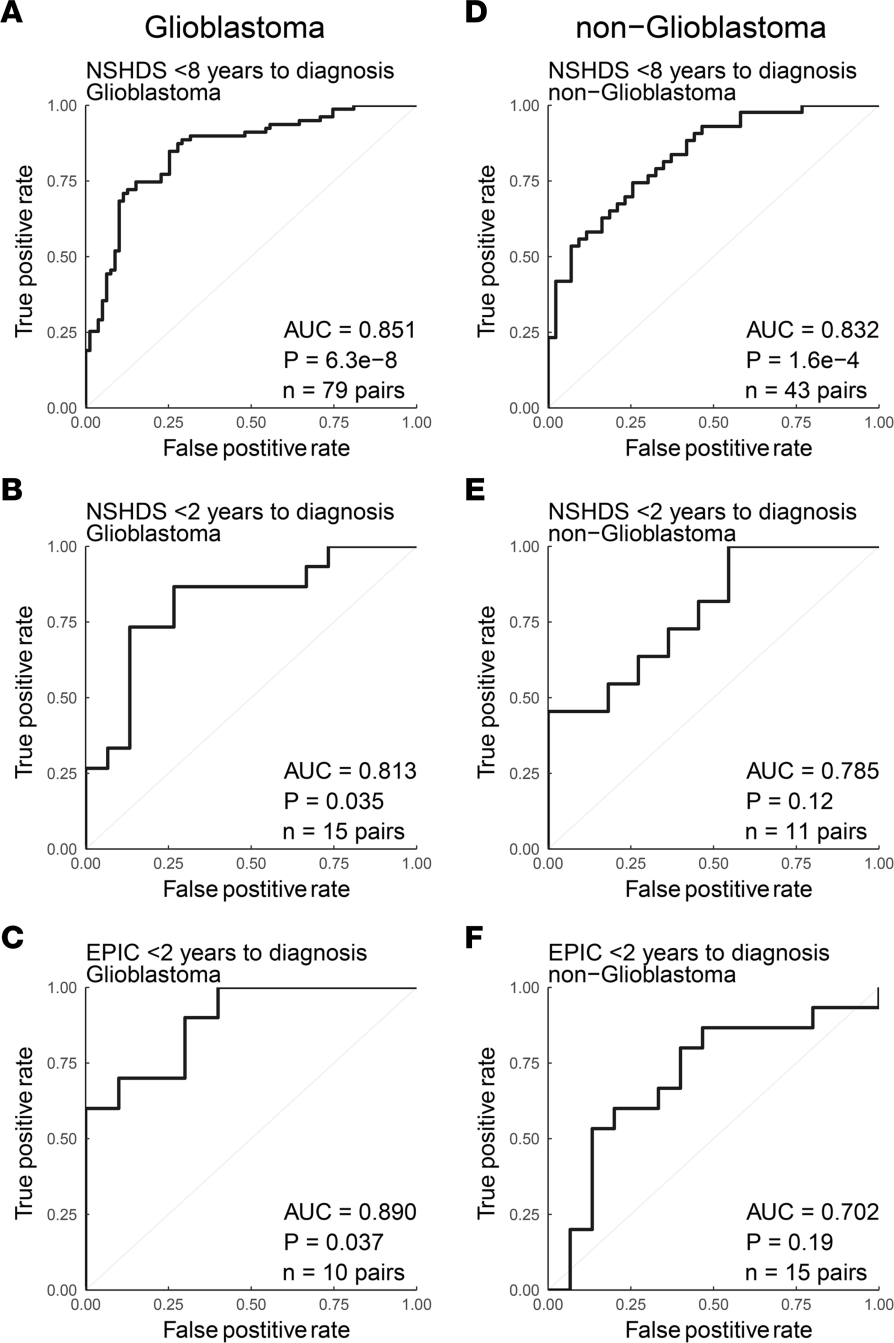
ROC analysis using a panel of 20 metabolites on glioma subtypes. (**A** and **B**) NSHDS glioblastoma case-control pairs sampled less than 8 years (**A**) or less than 2 years (**B**) before diagnosis. (**C**) EPIC glioblastoma case-control pairs sampled less than 2 years before diagnosis. (**D** and **E**) NSHDS nonglioblastoma case-control pairs sampled less than 8 years (**D**) or less than 2 years (**E**) before diagnosis. (**F**) EPIC nonglioblastoma case-control pairs sampled less than 2 years before diagnosis. Wilcoxon signed-rank test (2-sided) was used to calculate the significance of the ROC curves. *n* = number of pairs available for each analysis.

The blood metabolome is dynamic and affected by many exogenous and biological factors, highlighting the need to minimize confounding variation by study design. Since metabolic differences between males and females are obvious in blood samples, we wanted to assess our strategy and the predictive ability of the 20-metabolite panel on females and males separately. Also, here the panel predicted both female and male case-control pairs in NSHDS well, with AUC values for females of 0.870 and *P* = 3.4 × 10^–9^ (Figure 5A) and AUC values for males of 0.818 and *P* = 2.1 × 10^–4^ (Figure 5D). Prediction limited to case-control pairs within 2 years to diagnosis in NSHDS and EPIC also displayed solid AUC values for both females and males (Figure 5, B–F), with the best prediction of males in EPIC within 2 years of diagnosis (AUC = 0.964, *P* = 6.1 × 10^–4^). To further assess the predictive ability of the panel on different glioma subtypes, ROC analyses were performed on glioblastoma and all other gliomas (nonglioblastoma) separately. Case-control pairs within 8 and 2 years to diagnosis from NSHDS and within 2 years from EPIC were predicted (Figure 6). The panel performed well and gave slightly better predictions for glioblastoma, with AUCs of 0.851 and 0.813 in NSHDS within 8 and 2 years, respectively, and an AUC of 0.890 in EPIC within 2 years to diagnosis (Figure 6, A–C). Predictions of nonglioblastoma were also good, with AUCs of 0.832 and 0.785 in NSHDS within 8 and 2 years, respectively, and an AUC of 0.702 in EPIC within 2 years to diagnosis (Figure 6, D–F). However, the predictions of nonglioblastoma within 2 years in NSHDS and EPIC did not reach statistical significance, likely due to small sample sizes.

Due to coherent results of the detection of glioma development within 2 years to diagnosis in NSHDS and EPIC, we calculated the significance for metabolites within 2 years to diagnosis in case-control pairs from both cohorts. Seventeen of the 93 significant metabolites within 8 years to diagnosis were still significant within 2 years to diagnosis in NSHDS (Supplemental Figure 2A and Supplemental Table 3), whereas 3 were found significant within 2 years to diagnosis in EPIC (Supplemental Figure 2B and Supplemental Table 4).

### Altered metabolic pathways.

**Figure 7:**
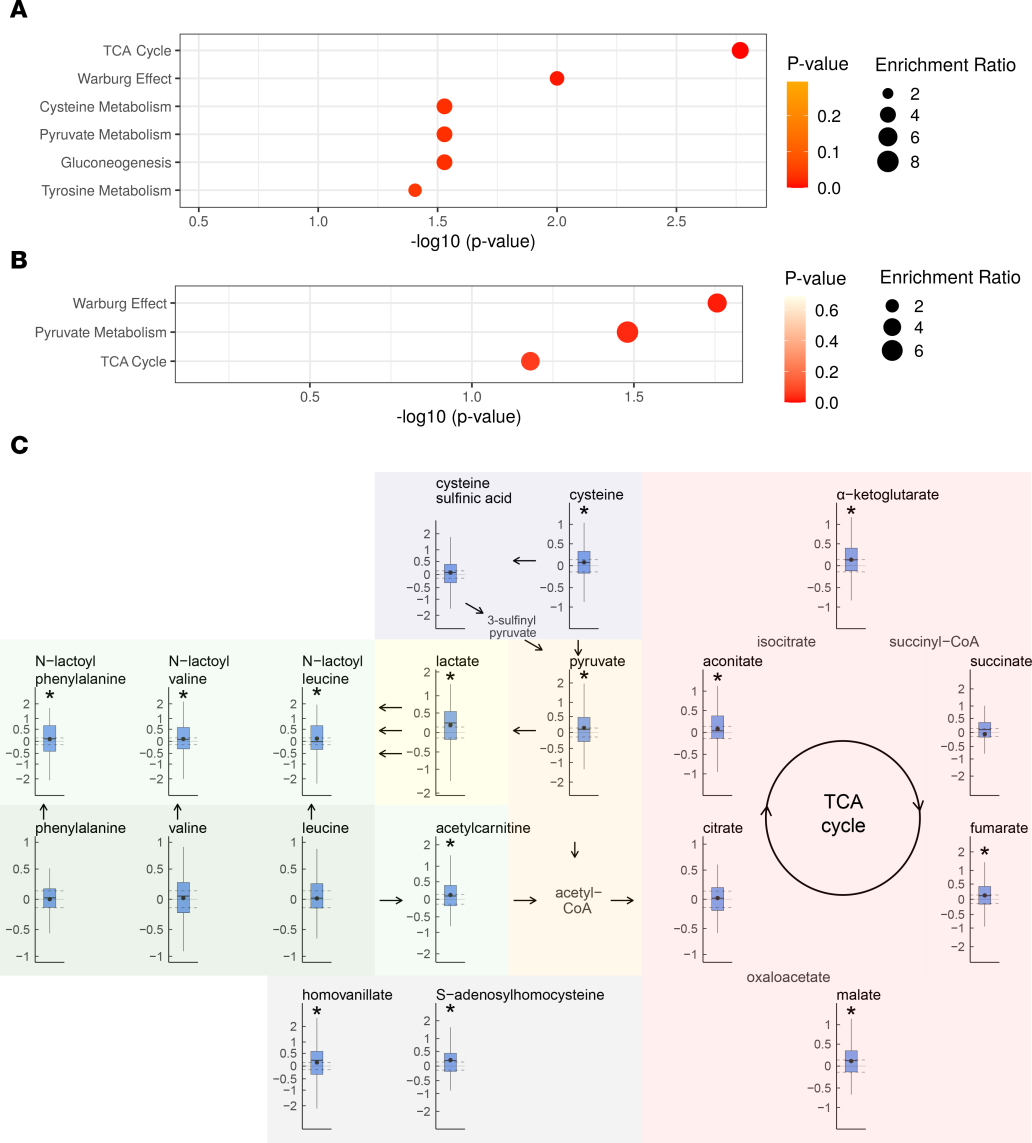
Overview of significant metabolic pathways. (**A** and **B**) Pathway enrichment analysis using the 93 metabolites significant within 8 years to diagnosis in NSHDS (**A**) and the panel of 20 metabolites in common for NSHDS and EPIC (**B**). A hypergeometric test was used to calculate significance (1 sided). (**C**) Detected metabolites present in the TCA cycle; the Warburg effect; gluconeogenesis; pyruvate, cysteine, and tyrosine metabolism; and neighboring amino acid metabolism. Box plots with average (dot) and median (line) log_2_ fold change are presented from case-control pairs within 8 years to diagnosis from NSHDS (*n* = 130). Dashed horizontal lines display a 10% difference. Significant metabolites, calculated by multivariate significance (2-sided), are denoted as *P < 0.05. Undetected pathway metabolites are included with name without box plot.

We performed a metabolite enrichment analysis to put the panel of 20 metabolites in common and the 93 significant metabolites discovered in NSHDS into biological context. For the 93 significant metabolites, the most significant overrepresented metabolic pathways were the TCA cycle (*P* = 0.002) and the Warburg effect (*P* = 0.01) (Figure 7A). Other significantly overrepresented pathways (*P* < 0.05) were pyruvate and cysteine metabolism, gluconeogenesis, and tyrosine metabolism. For the 20-metabolite panel, with consistent metabolite level differences in NSHDS and in EPIC closer to diagnosis, the Warburg effect (*P* = 0.02), pyruvate metabolism (*P* = 0.03), and the TCA cycle (*P* = 0.07) were still the most overrepresented pathways (Figure 7B). The metabolites within the significant pathways are however tightly connected. The significant metabolites and pathways, together with neighboring pathway of amino acid metabolism, are presented in Table 4 and Figure 7C. In this analysis, the levels of all significant metabolites within the presented pathways were higher in cases compared with controls. In addition, the levels were even higher toward diagnosis for all metabolites (Supplemental Figure 3).

Finally, we examined the plasma levels of 2-hydroxyglutarate as several endogenously expressed TCA cycle –related metabolites were found to be significantly altered. Plasma levels of 2-hydroxyglutarate, the oncometabolite produced by a mutation in isocitrate dehydrogenase, showed elevated levels closer to diagnosis in both NSHDS and EPIC samples (Supplemental Figure 4) but did not reach statistical significance. As isocitrate dehydrogenase mutation is uncommon in glioblastoma, we examined glioblastoma and nonglioblastoma cases separately (Supplemental Figure 4 and Supplemental Methods). Here, the plasma levels of 2-hydroxyglutarate followed the same trend as observed for all glioma combined, except for nonglioblastoma in EPIC samples that showed reduced levels toward diagnosis.

## Discussion

In this study, we found 93 metabolites in NSHDS with significantly different plasma levels within 8 years of glioma diagnosis compared with healthy controls. In addition, 20 of these metabolites displayed consistent metabolite-level differences closer to diagnosis in samples from the NSHDS cohort and the multicenter EPIC cohort, with a mean difference of > 10% between cases and controls. This panel of 20 metabolites showed good ability to separate future glioma cases from matched controls within 8 years to diagnosis in NSHDS and within 2 years to diagnosis in EPIC, independently of biological sex or glioma subtype. Our results are in line with previous studies that have detected metabolic alterations in prediagnostic plasma samples up to 8 years before glioma diagnosis (5) and longitudinal whole-genome profiling of gliomas showing that mutated founder cells with common genetic aberrations emerge up to 7 years before diagnosis (4). Metabolites in our panel have previously been linked to tumor metabolism, which in our view strengthens their validity. Our metabolite enrichment analysis particularly highlighted metabolites linked to the TCA cycle pathway and the Warburg effect as the most affected. Elevated plasma levels of fumarate and cystine were particularly robust in prediagnostic cases from both NSHDS and EPIC within 8 years to diagnosis.

The TCA cycle was found significantly overrepresented in the enrichment analyses. Elevated levels of TCA cycle–related metabolites, fumarate, succinate, and D-2-hydroxyglutarate, have previously been linked to oncometabolite-driven tumorigenesis (7). TCA cycle–related metabolites play a central role in the Warburg effect. The Warburg effect, observed in glioma cells and other cancers, is characterized by metabolic reprogramming causing an increased rate of glycolysis and production of lactate under aerobic conditions with functioning mitochondria (13, 14). Accumulated lactate is released from the cell and acidifies the tumor microenvironment, favoring tumor progression. Here, we report significantly elevated levels of lactate in prediagnostic glioma cases within 8 years to diagnosis. In addition, we found significantly elevated levels of N-lactoyl valine, N-lactoyl leucine, and N-lactoyl phenylalanine within 8 years to diagnosis. N-lactoyl amino acid production is catalyzed by reverse proteolysis of lactate and amino acids by carnosine dipeptidase 2 (15). N-lactoyl amino acids are poorly studied, and their role in glioma development and cancer is unknown. Interestingly, 7 of the 20 metabolites in our panel (lactate, fumarate, malate, hypoxanthine, N-lactoyl valine, N-lactoyl leucine, and N-lactoyl phenylalanine) are some of the most elevated metabolites in blood during physical activity (16, 17). Moreover, exercise-induced N-lactoyl phenylalanine has recently been hypothesized to function as a molecular signal to regulate energy balance (17). Hypothetically, the shared set of metabolites related to glioma development and physical activity may be linked to inflammatory mediators, since elevated levels of inflammatory cytokines have also been reported in prediagnostic glioma blood (18). Elevated levels of lactate and hypoxanthine have also been reported in blood of people with immune-mediated inflammatory disease (19). These metabolites may reflect a state of increased energy demand and energy turnover caused by inflammation.

In our analysis, products of the tyrosine metabolism were also found significant, with higher levels of homovanillate and S-adenosylhomocysteine and lower levels of tyramine O-sulfate in prediagnostic glioma cases. In the brain, tyrosine is the starting material for synthesis of catecholamines (20). Homovanillate is the end product of dopamine catabolism and is elevated in urine of patients with catecholamine-secreting tumors such as neuroblastoma (21). Altered tyrosine metabolism has previously also been found to be related to glioma development, where elevated plasma levels of 4-hydroxyphenylacetic acid were detected in prediagnostic glioma cases (5).

Our findings are also consistent with previous reports of imbalanced redox homeostasis for prediagnostic glioma cases, highlighting elevated levels of metabolites such as cystine, cysteine, eryhtritol, erythronate, and hypoxanthine (5, 6). However, a complete overlap and replication of significant metabolites between current and previous studies are not to be expected, as the analyses were performed on different analytical platforms with different metabolite coverages.

As stated, NSHDS and EPIC samples were collected using different blood anticoagulants and the majority of the participants have different fasting status between the cohorts, which — together with the multicenter structure of EPIC — introduced variation unrelated to the research question and complicated the validation of discovered metabolites in NSHDS. However, these differences also imply some degree of robustness to our findings, as they were consistent in 2 largely diverse cohorts.

Our results show that glioma development is detectable in blood up to 2 years before diagnosis and even up to 8 years before diagnosis in a homogenous sample population such as NSHDS. Other disease studies have shown that blood tests have the potential to detect neurological disorders, such as Parkinson’s and Alzheimer’s disease, in their early stages (22, 23). Clinically, a blood test for glioma diagnostics could be used for early detection in patients with nonspecific symptoms or to discriminate unclear lesions at brain imaging. The panel of 20 metabolites presented here shows potential to serve as a diagnostic tool, and future studies should target these metabolites in a clinical setting, in patients with nonspecific symptoms and those with other cancer types, to evaluate their specificity toward glioma. Furthermore, the altered plasma metabolite levels are not proven here to be the result of glioma cancer cells, since the altered metabolite levels can equally be a result of cells in the microenvironment or just an altered metabolism throughout the body as a consequence of disease progression. We recently showed that WHO-classified subtypes of glioma tumors have different metabolic phenotypes that reach beyond isocitrate dehydrogenase (IDH) mutation status (24). A question that remains to be answered is whether blood-based metabolomics can differentiate various molecular subtypes. Although we anticipate that our findings will greatly help elucidate the mechanism of gliomagenesis and to find therapeutic targets, affected metabolic and biochemical pathways are still to be fully characterized before clinical applications can be developed.

## Methods

### Study population and nested case-control design.

We conducted a nested case-control study within 2 population-based prospective cohorts, NSHDS and EPIC. Detailed information about the cohorts is given in Supplemental Methods. Incident glioma cases in NSHDS (International Classification of Diseases-7 [ICD-7], topography: 193, histology: 475–476) and EPIC (ICD-O-2, topography: C71, histology: 93800–94800) were identified via cancer registries or through active follow up. Each case was randomly paired with a matching control that, at the time of diagnosis of the index case, was alive and free of cancer (except nonmelanoma skin cancer). Matching was based on sex, BMI, age (± 6 months), fasting status, time of sampling (± 3 months in NSHDS and ± 1 month in EPIC), and study center. In total, 1,102 blood samples were included: 528 EDTA-plasma samples (264 prediagnostic glioma case samples and 264 control samples) from NSHDS and 574 sodium citrate plasma samples (287 prediagnostic glioma cases and 287 controls) from EPIC. The EPIC samples were from Spain, Italy, United Kingdom, the Netherlands, Germany, and Norway. Additional information regarding the blood samples is given in Supplemental Methods. In this study, we used samples from the single-center NSHDS cohort for discovery and the multicenter EPIC cohort for validation.

### Metabolomics analyses.

Metabolite analysis and data curation are described in detail in Supplemental Methods. We designed a constrained randomized run order (25) — i.e., each case-control pair was run directly adjacent to each other in randomized order. All samples were analyzed using the Metabolon Inc. global metabolomics platform, consisting of 4 untargeted ultra high–performance LC–MS/MS (UHPLC-MS/MS) methods.

For targeted quantitative measurements of lactate, we used the LC-MS/MS–based Biocrates MxP500 quant platform and analyzed 354 NSHDS samples. This analysis is described in detail in Supplemental Methods.

### Statistics.

We analyzed matched case-control pairs as dependent samples throughout the study. For this purpose, an effect matrix with differences of relative concentrations for each metabolite of a case and its matched control was constructed. All statistical tests were 2 sided, except for the 1-sided hypergeometric test used in the metabolite enrichment analysis (Figure 7, A and B, and Table 4). *P* < 0.05 was considered significant for all tests.

To get an overview of the samples, we performed Principal Component Analysis (PCA) (26) on case-control pairs from NSHDS and EPIC separately. One extreme outlier sample pair was observed in the PCA of NSHDS that indicated an abnormal plasma concentration difference within the pair, and it was excluded from further data analysis, resulting in a final number of 550 cases and 550 controls. Furthermore, to get an overview of samples from both cohorts simultaneously, UMAP analysis was performed. UMAP plots were constructed for all samples, as individual observations, and for sample pairs using the effect matrix with calculated differences of matched case-control pairs.

To discover metabolites indicating glioma development, we performed multivariate modeling using OPLS-EP (25) with the effect matrix of case-control pairs from NSHDS and the curated metabolomics data of 1,061 molecular features (Supplemental Methods). Significance of the OPLS-EP model was calculated using CV-ANOVA (2 sided) (27). Only metabolites in NSHDS that were multivariate significant (2-sidedmultivariate significance test) (5, 28) were selected for validation in EPIC. For validation, the difference between cases and controls in metabolite levels toward diagnosis of the significant metabolites were examined in both NSHDS and EPIC. Metabolites that displayed the same direction toward diagnosis, with a mean difference of > 10% closer to diagnosis (within 2 years and/or 1 year to diagnosis), were identified and examined on their ability to detect glioma development. The results were evaluated with ROC analyses. We calculated the AUC and the significance of the ROC curves using the Wilcoxon signed-rank test (2 sided). To assess if predictions were deviating depending on biological sex or glioma subtype, ROC analyses were done for females and males separately and for all glioma, glioblastoma, and nonglioblastoma separately (Supplemental Methods).

To put metabolites into biological context and to find altered metabolic pathways, we performed metabolite enrichment analysis using Metaboanalyst 5.0 (www.metaboanalyst.ca). For this analysis, we included metabolites within the curated NSHDS data set — in total, 736 identified metabolites — with known HMDB ID that were coherent with the Metaboanalyst database as a reference library. A hypergeometric test was used to calculate significance (1 sided).

### Study approval.

The IRB of the IARC and the local ethics committees at each study center approved the study. All participants provided written informed consent. All samples were pseudonymized and included in the study in accordance with the ethical standards of the Declaration of Helsinki. This project was approved by the ethical review board at Umeå University (Dnr 2017-295-31M).

### Data availability.

Data values associated with the manuscript and supplemental material shown in graphs are presented in the Supporting Data Values. The complete data sets generated for these analyses will be shared upon request to the corresponding authors. Data access requires ethical approval, as existing informed consent will not permit personal data to be shared publicly. Requests will be reviewed by representatives of the NSHDS/EPIC steering committee.

## Author contributions

Conceptualization was contributed by BM and BB; resources were contributed by UA, BM, RK, MBS, VP, IU, PA, SMCY, MG, AKH, ACC, and MJ; methodology was contributed by SL, BB, and BM; data curation was contributed by SL and BB; formal analysis was contributed by SL and BB; validation was contributed by SL and BB; interpretation of results was contributed by SL, BB, BM, and HA; visualizations were contributed by SL; writing of the original draft was contributed by SL and BB; review and editing of the manuscript were contributed by SL, BB, BM, HA, and LN; and input and valuable comments were contributed by RK, MBS, VP, IU, PA, SMCY, MG, AKH, ACC, and MJ. All authors read and approved the final manuscript.

## Supplementary Material

Supplemental data

Supporting data values

## Figures and Tables

**Table 4:**
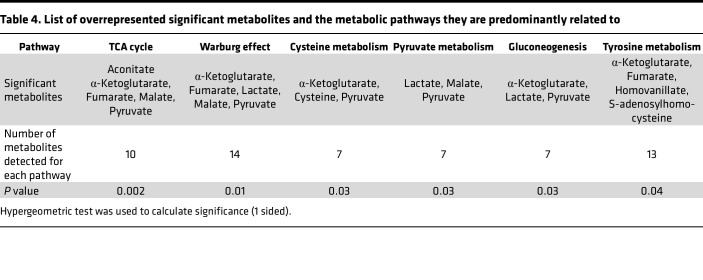
List of overrepresented significant metabolites and the metabolic pathways they are predominantly related to

**Table 3:**
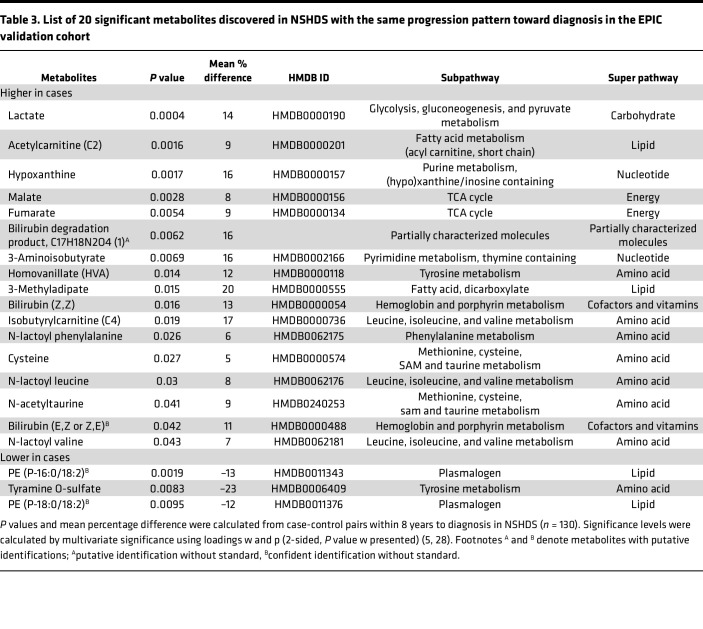
List of 20 significant metabolites discovered in NSHDS with the same progression pattern toward diagnosis in the EPIC validation cohort

**Table 2:**
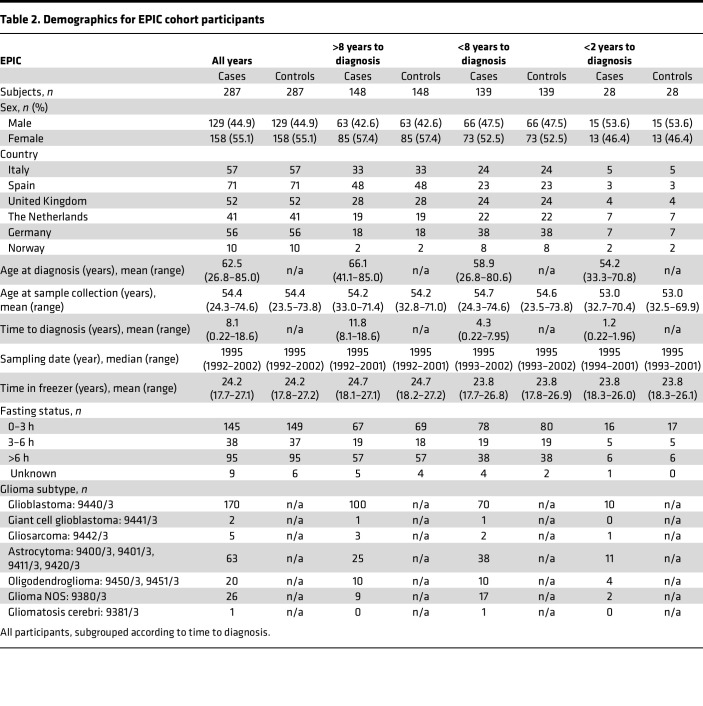
Demographics for EPIC cohort participants

**Table 1:**
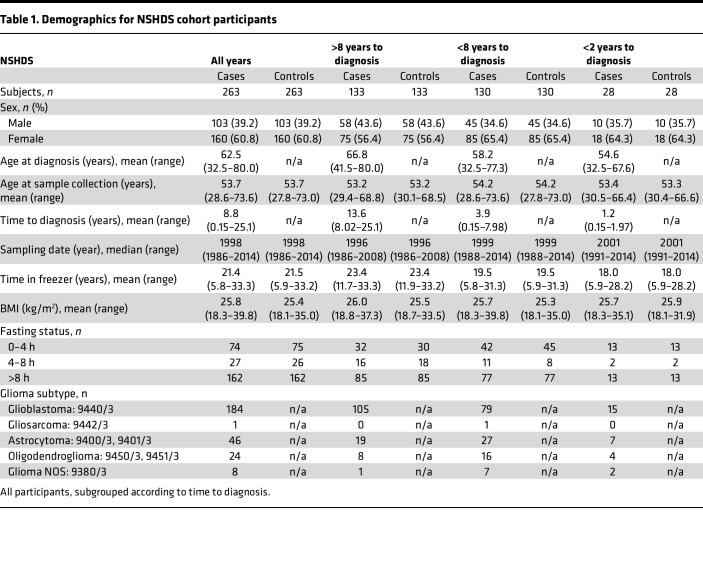
Demographics for NSHDS cohort participants

## Version 1 08/31/2023

In-Press Preview

## Version 2 10/09/2023

Electronic publication
