# Supplementary material for: Altered plasma metabolite levels can be detected years before a glioma diagnosis
Source: JCI Insight. 2023 Oct 9;8(19):e171225. doi: 10.1172/jci.insight.171225 (PMC10619434; doi:10.1172/jci.insight.171225)
Supplement: Supplemental data [file jciinsight-8-171225-s063.pdf]

Supplemental Methods

Study Population and Nested Case-Control Design

We conducted a retrospective nested case-control study within two population-based prospective cohorts, NSHDS and EPIC. NHSDS is an ongoing cohort in Västerbotten County, Sweden with over 135 000 participants (1, 2). Participants were invited to donate blood samples, which thereafter were stored at the Northern Sweden Biobank. EPIC enrolled over 521 000 participants during 1992-1999 from 23 centers in ten European countries (3). Details of subject recruitment, study design, blood collection protocol and follow-up procedure have been published elsewhere (1, 3, 4).

Matched case-control pairs that had opposite fasting status, suspected hemolyzed sample, or mistakenly been separated into different analytical batches by the platform, were not included. The 264 samples from pre-diagnostic cases in NSHDS were mostly collected at health examinations at the age of 40, 50 and 60, originating from 163 different individuals who had donated 1-3 blood samples over several years, with an average of over seven years between first and last sample collections. A set of these NSHDS samples was used in our previous study (5), analyzed then on a different platform.

Metabolomics analysis

The run order was balanced to achieve an even distribution of glioma subtype diagnosis, sex, age, and time of sampling. The samples from the NSHDS and EPIC cohorts were analyzed separately since different anticoagulants were used during sampling, causing the cohorts to have different sample matrices. We incorporated

quality control measures by including blanks samples, pooled quality control plasma samples and serial dilutions throughout the run (6).

The platform consists of four Ultrahigh Performance Liquid Chromatography-Tandem Mass Spectroscopy (UHPLC-MS/MS) methods, each optimized to detect different types of metabolites. Two methods with reverse phase (RP) UHPLC-MS/MS using positive ion mode electrospray ionization (ESI) chromatographically optimized for hydrophilic metabolites and for hydrophobic metabolites, respectively. Another RP-UHPLC-MS/MS method in negative ion mode ESI, and a hydrophilic interaction liquid chromatography UHPLC-MS/MS method using negative ion mode ESI. All four methods were run to detect a wide range of metabolites. Detailed information of the procedures for metabolite extraction, quality assurance, metabolite analysis and data processing have been extensively described elsewhere (7).

The data was curated prior to statistical analysis. In NSHDS, 1374 molecular features were quantified, out of which 1095 metabolites were identified. In EPIC, 1214 molecular features were quantified, where 1016 metabolites were identified. In total, 1094 molecular features and 915 identified metabolites were common for samples from both cohorts before data curation. Serial dilutions of pooled quality control reference samples were used to exclude molecular features not correlating with a linear quantification, as determined by calculating coefficient of determination values. To minimize the influence of instrument drift, raw peak area data was batch-normalized, i.e. each molecular feature in each sample batch was divided by the batch median detection level of that molecular feature. For multivariate statistical analysis, missing values were imputed half of the minimum intensity detected of that metabolite. The robustness of the analysis was assessed by examination of relative

standard deviation (RSD) for the detected metabolites in our study-specific pooled plasma samples and reported RSDs from Metabolon. Metabolites with more than 20% missing values, in both cases and controls, were excluded from statistical analysis (8, 9). Median RSD from our pooled plasma samples was 15.7% and 13.8% in NSHDS and EPIC, respectively, and over 90% of the molecular features had an RSD below 30%. After completed data curation, 1061 molecular features with 856 identified metabolites remained for NSHDS, and 875 molecular features and 751 identified metabolites for EPIC. In total, 802 molecular features and 691 metabolites were in common for both NSHDS and EPIC.

For targeted quantitative measurements of lactate, we used the LC-MS/MS-based Biocrates MxP500 quant platform and analyzed 354 of NSHDS samples. This targeted analysis was done in-house according to the manufactures protocol using a triple Quad UPLC Waters Xevo TQ-XS system and the MetIDQ software. A linear regression model was calculated based on the relative lactate levels from Metabolon and the quantitative lactated levels from the Biocrates MxP500. Lactate levels of NSHDS samples that were not measured quantitatively were then predicted using the linear regression model. Significance and lactate levels in case-control pairs towards diagnosis were compared between the analytical measurements.

Statistics

Statistical analyses were performed using R (version 4.1.3) and MATLAB (R2020b, update 1), and OPLS-EP and PCA models were generated using SIMCA (version 16.0, Sartorius Stedim Data Analytics AB).

To discover metabolites indicating glioma development, we performed multivariate modeling using Orthogonal Projections to Latent Structures – Effect Projection (OPLS-EP) (10) with the effect matrix of case-control pairs from NSHDS. The curated metabolomics data was used with the 1061 molecular features as X (input) variables and scaled to unit variance without centering. The Y (response) variable, a vector of ones, was not scaled. Selection of number of OPLS model components was based on the lowest obtained CV-ANOVA p-value. This approach reduces the risk of overfitting the models as P-values are penalized by increasing number of components. Variable selection was based on multivariate significance with a threshold of $\alpha=0.05$ (5, 11).

Metabolites with the same progression pattern towards diagnosis in NSHDS and EPIC were examined on their ability to detect glioma development. The results were evaluated with ROC analyses. We calculated AUC and we used Wilcoxon signed-rank test to calculate the significance of the ROC curves. To assess if predictions were deviating depending on glioma subtype, ROC analyses were done besides for all glioma also for glioblastoma and non-glioblastoma, separately. To have a clear division between glioblastoma and non-glioblastoma, subtypes of glioblastoma (9441/3, 9442/3, n=2) and cases with insufficient subtype information (9380/3, n=9) were excluded in the ROC analyses.

The same division of glioblastoma and non-glioblastoma was made for the analysis of 2-hydroxyglutarate levels towards diagnosis in NSHDS and EPIC, where glioblastoma subtypes (9441/3, 9442/3, n=8) and glioma cases with insufficient subtype information (9380/3, 9381/3, n=35) were excluded.

References

1. Hallmans G, et al. Cardiovascular disease and diabetes in the Northern Sweden Health and Disease Study Cohort - evaluation of risk factors and their interactions. *Scand J Public Healt*. 2003;31:18-24.
2. . Nothern Sweden Health and Disease Study, NSHDS. https://www.umu.se/en/biobank-research-unit/provsamlingar-och-register/northern-sweden-health-and-disease-study-vip-monica-and-the-mammography-screening-project/ Accessed June 17, 2022.
3. Riboli E, et al. European prospective investigation into cancer and nutrition (EPIC): study populations and data collection. *Public Health Nutr*. 2002;5(6b):1113-24.
4. Riboli E, and Kaaks R. The EPIC project: Rationale and study design. *Int J Epidemiol*. 1997;26:S6-S14.
5. Jonsson P, et al. Identification of Pre-Diagnostic Metabolic Patterns for Glioma Using Subset Analysis of Matched Repeated Time Points. *Cancers*. 2020;12(11).
6. Dudzik D, et al. Quality assurance procedures for mass spectrometry untargeted metabolomics. a review. *J Pharmaceut Biomed*. 2018;147:149-73.
7. Pietzner M, et al. Plasma metabolites to profile pathways in noncommunicable disease multimorbidity. *Nat Med*. 2021;27(3):471-9.
8. Smilde AK, et al. Fusion of mass spectrometry-based metabolomics data. *Anal Chem*. 2005;77(20):6729-36.
9. Yang J, et al. A data preprocessing strategy for metabolomics to reduce the mask effect in data analysis. *Front Mol Biosci*. 2015;2.
10. Jonsson P, et al. Constrained randomization and multivariate effect projections improve information extraction and biomarker pattern discovery in metabolomics studies involving dependent samples. *Metabolomics*. 2015;11(6):1667-78.
11. Jonsson P, et al. Statistical loadings and latent significance simplify and improve interpretation of multivariate projection models. *bioRxiv*. 2018.

Supplemental Tables

Supplemental Table 1. Mutlivariate significant metabolites within eight years to diagnosis in NSHDS (n=130 pairs).

Metabolite	P-value	Mean % difference	HMDB ID	Sub-pathway	Super pathway
Higher in cases					
**N-carbamoylvaline**	0.00002	25		Leucine, Isoleucine and Valine Metabolism	Amino Acid
**N2,N2-dimethylguanosine**	0.00008	9	HMDB0004824	Purine Metabolism, Guanine containing	Nucleotide
**alpha-ketoglutarate**	0.00038	10	HMDB0000208	TCA Cycle	Energy
**lactate**	0.00039	14	HMDB0000190	Glycolysis, Gluconeogenesis, and Pyruvate Metabolism	Carbohydrate
**N-acetylalanine**	0.00051	6	HMDB0000766	Alanine and Aspartate Metabolism	Amino Acid
**acetylcarnitine (C2)**	0.0016	9	HMDB0000201	Fatty Acid Metabolism (Acyl Carnitine, Short Chain)	Lipid
**androstenediol (3beta,17beta) monosulfate (2)**	0.0016	24	HMDB0186954	Androgenic Steroids	Lipid
**succinoyltaurine**	0.0016	16		Methionine, Cysteine, SAM and Taurine Metabolism	Amino Acid
**hypoxanthine**	0.0017	16	HMDB0000157	Purine Metabolism, (Hypo)Xanthine/Inosine containing	Nucleotide
**N-acetylvaline**	0.0017	7	HMDB0011757	Leucine, Isoleucine and Valine Metabolism	Amino Acid
**C-glycosyltryptophan**	0.0024	6	HMDB0240296	Tryptophan Metabolism	Amino Acid
**S-adenosylhomocysteine (SAH)**	0.0024	20	HMDB0000939	Methionine, Cysteine, SAM and Taurine Metabolism	Amino Acid
**dimethylarginine (SDMA + ADMA)**	0.0024	5	HMDB0001539	Urea cycle; Arginine and Proline Metabolism	Amino Acid
**malate**	0.0028	8	HMDB0000156	TCA Cycle	Energy
**N-acetylserine**	0.0028	5	HMDB0002931	Glycine, Serine and Threonine Metabolism	Amino Acid
**tetradecadienedioate (C14:2-DC)***	0.0031	23		Fatty Acid, Dicarboxylate	Lipid
**3-(3-amino-3-carboxypropyl)uridine***	0.0033	8	HMDB0242130	Pyrimidine Metabolism, Uracil containing	Nucleotide
**dihomo-linolenoylcarnitine (C20:3n3 or 6)***	0.0034	16		Fatty Acid Metabolism (Acyl Carnitine, Polyunsaturated)	Lipid
**ximenoylcarnitine (C26:1)***	0.0036	10		Fatty Acid Metabolism (Acyl Carnitine, Monounsaturated)	Lipid
**5-methylthioadenosine (MTA)**	0.0036	7	HMDB0001173	Polyamine Metabolism	Amino Acid
**orotidine**	0.0043	7	HMDB0000788	Pyrimidine Metabolism, Orotate containing	Nucleotide
**N6-acetyllysine**	0.0047	7	HMDB0000206	Lysine Metabolism	Amino Acid
**arachidonoylcarnitine (C20:4)**	0.0048	15	HMDB0006455	Fatty Acid Metabolism (Acyl Carnitine, Polyunsaturated)	Lipid

**ribonate**	0.0048	7	HMDB0000867	Pentose Metabolism	Carbohydrate
**fumarate**	0.0054	9	HMDB0000134	TCA Cycle	Energy
**gulonate***	0.0054	19	HMDB0003290	Ascorbate and Aldarate Metabolism	Cofactors and Vitamins
**pyruvate**	0.0057	10	HMDB0000243	Glycolysis, Gluconeogenesis, and Pyruvate Metabolism	Carbohydrate
**erythronate***	0.0058	5	HMDB0000613	Aminosugar Metabolism	Carbohydrate
**androstenediol (3beta,17beta) disulfate (1)**	0.0061	17	HMDB0240313	Androgenic Steroids	Lipid
**bilirubin degradation product, C17H18N2O4 (1)****	0.0062	16		Partially Characterized Molecules	Partially Characterized Molecules
**erythritol**	0.0064	11	HMDB0002994	Food Component/Plant	Xenobiotics
**3-aminoisobutyrate**	0.0069	16	HMDB0002166	Pyrimidine Metabolism, Thymine containing	Nucleotide
**hydroxyasparagine****	0.0071	6	HMDB0032332	Alanine and Aspartate Metabolism	Amino Acid
**dimethyl sulfone**	0.0075	16	HMDB0004983	Chemical	Xenobiotics
**decadienedioic acid (C10:2-DC)****	0.0081	31	HMDB0242172	Fatty Acid, Dicarboxylate	Lipid
**citrulline**	0.0086	7	HMDB0000904	Urea cycle; Arginine and Proline Metabolism	Amino Acid
**5alpha-androstan-3beta,17beta-diol disulfate**	0.0088	13	HMDB0000493	Androgenic Steroids	Lipid
kynurenine	0.010	7	HMDB0000684	Tryptophan Metabolism	Amino Acid
cortolone glucuronide (1)	0.011	14	HMDB0010320	Corticosteroids	Lipid
N6-carbamoylthreonyladenosine	0.012	5	HMDB0041623	Purine Metabolism, Adenine containing	Nucleotide
(R)-3-hydroxybutyrylcarnitine	0.013	13	HMDB0013127	Fatty Acid Metabolism (Acyl Carnitine, Hydroxy)	Lipid
N1-methyladenosine	0.013	4	HMDB0003331	Purine Metabolism, Adenine containing	Nucleotide
homovanillate (HVA)	0.014	12	HMDB0000118	Tyrosine Metabolism	Amino Acid
N-acetylisoleucine	0.015	8	HMDB0061684	Leucine, Isoleucine and Valine Metabolism	Amino Acid
3-methyladipate	0.015	20	HMDB0000555	Fatty Acid, Dicarboxylate	Lipid
7-alpha-hydroxy-3-oxo-4-cholestenoate (7-Hoca)	0.016	7	HMDB0012458	Sterol	Lipid
bilirubin (Z,Z)	0.016	13	HMDB0000054	Hemoglobin and Porphyrin Metabolism	Cofactors and Vitamins
gamma-glutamylcitrulline*	0.017	11		Gamma-glutamyl Amino Acid	Peptide
aconitate [cis or trans]	0.018	6	HMDB0000072	TCA Cycle	Energy
indoleacetylglutamine	0.019	13	HMDB0013240	Tryptophan Metabolism	Amino Acid
isobutyrylcarnitine (C4)	0.019	17	HMDB0000736	Leucine, Isoleucine and Valine Metabolism	Amino Acid
2,3-dihydroxy-5-methylthio-4-pentenoate (DMTPA)*	0.020	5	HMDB0240388	Methionine, Cysteine, SAM and Taurine Metabolism	Amino Acid
mannonate*	0.021	9	HMDB0242119	Food Component/Plant	Xenobiotics

N-acetylputrescine	0.022	6	HMDB0002064	Polyamine Metabolism	Amino Acid
sphingosine 1-phosphate	0.023	7	HMDB0000277	Sphingosines	Lipid
hypotaurine	0.023	10	HMDB0000965	Methionine, Cysteine, SAM and Taurine Metabolism	Amino Acid
N,N,N-trimethyl-5-aminovalerate	0.024	10	HMDB0240732	Lysine Metabolism	Amino Acid
malonylcarnitine	0.025	26	HMDB0002095	Fatty Acid Synthesis	Lipid
N-lactoyl phenylalanine	0.026	6	HMDB0062175	Phenylalanine Metabolism	Amino Acid
cysteine	0.027	5	HMDB0000574	Methionine, Cysteine, SAM and Taurine Metabolism	Amino Acid
3-hydroxy-3-methylglutarate	0.028	9	HMDB0000355	Mevalonate Metabolism	Lipid
cystine	0.029	13	HMDB0000192	Methionine, Cysteine, SAM and Taurine Metabolism	Amino Acid
N-lactoyl leucine	0.030	8	HMDB0062176	Leucine, Isoleucine and Valine Metabolism	Amino Acid
oleoylcarnitine (C18:1)	0.031	6	HMDB0005065	Fatty Acid Metabolism (Acyl Carnitine, Monounsaturated)	Lipid
N,N-dimethyl-pro-pro	0.032	4		Modified Peptides	Peptide
bilirubin degradation product, C17H20N2O5 (1)**	0.033	15		Partially Characterized Molecules	Partially Characterized Molecules
2R,3R-dihydroxybutyrate	0.033	10	HMDB0000498	Fatty Acid, Dihydroxy	Lipid
N-acetyl-beta-alanine	0.035	7	HMDB0061880	Pyrimidine Metabolism, Uracil containing	Nucleotide
pseudouridine	0.038	3	HMDB0000767	Pyrimidine Metabolism, Uracil containing	Nucleotide
biliverdin	0.039	14	HMDB0001008	Hemoglobin and Porphyrin Metabolism	Cofactors and Vitamins
dihomo-linoleoylcarnitine (C20:2)*	0.039	14	HMDB0240747	Fatty Acid Metabolism (Acyl Carnitine, Polyunsaturated)	Lipid
isocitric lactone	0.039	12		TCA Cycle	Energy
N-acetyltaurine	0.041	9	HMDB0240253	Methionine, Cysteine, SAM and Taurine Metabolism	Amino Acid
formiminoglutamate	0.042	10	HMDB0000854	Histidine Metabolism	Amino Acid
bilirubin (E,Z or Z,E)*	0.042	11	HMDB0000488	Hemoglobin and Porphyrin Metabolism	Cofactors and Vitamins
N-lactoyl valine	0.043	7	HMDB0062181	Leucine, Isoleucine and Valine Metabolism	Amino Acid
linoleoylcarnitine (C18:2)*	0.045	7	HMDB0006469	Fatty Acid Metabolism (Acyl Carnitine, Polyunsaturated)	Lipid
3-hydroxysebacate	0.047	11	HMDB0000350	Fatty Acid, Monohydroxy	Lipid
2-hydroxyphenylacetate	0.047	14	HMDB0000669	Phenylalanine Metabolism	Amino Acid
N2-acetyl,N6,N6-dimethyllysine	0.049	28		Lysine Metabolism	Amino Acid
Lower in cases					
**1-(1-enyl-palmitoyl)-2-linoleoyl-GPC (P-16:0/18:2)***	0.0011	-12	HMDB0011211	Plasmalogen	Lipid
**PE(P-16:0/18:2)***	0.0019	-13	HMDB0011343	Plasmalogen	Lipid

**propyl 4-hydroxybenzoate sulfate**	0.0022	-108	HMDB0240718	Benzoate Metabolism	Xenobiotics
**1-(1-enyl-palmitoyl)-2-oleoyl-GPE (P-16:0/18:1)***	0.0032	-12	HMDB0011342	Plasmalogen	Lipid
**1-linoleoyl-GPC (18:2)**	0.0060	-10	HMDB0010386	Lysophospholipid	Lipid
**tyramine O-sulfate**	0.0083	-23	HMDB0006409	Tyrosine Metabolism	Amino Acid
**PE(P-18:0/18:2)***	0.0095	-12	HMDB0011376	Plasmalogen	Lipid
4-oxo-retinoic acid	0.017	-27	HMDB0006285	Vitamin A Metabolism	Cofactors and Vitamins
sphingomyelin (d18:2/23:1)*	0.026	-6	HMDB0240668	Sphingomyelins	Lipid
sphingomyelin (d18:2/23:0, d18:1/23:1, d17:1/24:1)*	0.026	-12		Sphingomyelins	Lipid
1-(1-enyl-stearoyl)-2-oleoyl-GPE (P-18:0/18:1)	0.034	-9	HMDB0011375	Plasmalogen	Lipid
tartronate (hydroxymalonate)	0.035	-8	HMDB0035227	Food Component/Plant	Xenobiotics
1,2-dilinoleoyl-GPC (18:2/18:2)	0.049	-7	HMDB0008138	Phosphatidylcholine (PC)	Lipid

Significance was calculated by multivariate significance (two-sided, P-value w presented). Metabolites in bold were also significant after using Benjamini-Hochberg procedure for controlling false discovery rate of 0.2. Identities of metabolites denoted with * or ** have not been confirmed using standards. The identity were still assigned with confidence for metabolites denoted with * and with reasonable confidence for metabolites denoted with **.

Supplemental Table 2. Mutlivariate significant metabolites more than eight years to diagnosis in the NSHDS cohort (n=133 pairs).

Metabolite	NSHDS		EPIC		HMDB ID	Sub-pathway	Super Pathway
	P-value	Mean % difference	P-value	Mean % difference			
Higher in cases							
**4-hydroxyglutamate**	0.0015	36	0.080[a]	-20	HMDB0002273	Glutamate Metabolism	Amino Acid
**N-acetylglutamate**	0.0020	11			HMDB0001138	Glutamate Metabolism	Amino Acid
homoarginine	0.011	9			HMDB0000670	Urea cycle; Arginine and Proline Metabolism	Amino Acid
glycochenodeoxycholate glucuronide (1)	0.012	20			HMDB0002579	Primary Bile Acid Metabolism	Lipid
3-ureidopropionate	0.015	10			HMDB0000026	Pyrimidine Metabolism, Uracil containing	Nucleotide
caprylate (8:0)	0.027	11			HMDB0000482	Medium Chain Fatty Acid	Lipid
N-acetyl-isoputreanine	0.028	7			HMDB0094713	Polyamine Metabolism	Amino Acid
2-hydroxypalmitate	0.028	6			HMDB0031057	Fatty Acid, Monohydroxy	Lipid
taurochenodeoxycholic acid 3-sulfate	0.030	16			HMDB0002486	Secondary Bile Acid Metabolism	Lipid
5-hydroxyhexanoate	0.039	1			HMDB0000525	Fatty Acid, Monohydroxy	Lipid
succinoyltaurine	0.042	17				Methionine, Cysteine, SAM and Taurine Metabolism	Amino Acid
citramalate	0.047	16			HMDB0000426	Glutamate Metabolism	Amino Acid
(N(1) + N(8))-acetylspermidine	0.049	6			HMDB0001276	Polyamine Metabolism	Amino Acid
Lower in cases							
**tiglylcarnitine (C5:1-DC)**	0.00002	-17	0.097	-6	HMDB0002366	Leucine, Isoleucine and Valine Metabolism	Amino Acid
**2-aminobutyrate**	0.00003	-12			HMDB0000452	Glutathione Metabolism	Amino Acid
**alpha-ketobutyrate**	0.00021	-37			HMDB0000005	Methionine, Cysteine, SAM and Taurine Metabolism	Amino Acid
**1-palmitoyl-2-docosahexaenoyl-GPE (16:0/22:6)***	0.00023	-25			HMDB0008946	Phosphatidylethanolamine (PE)	Lipid
**N-palmitoyl-sphingosine (d18:1/16:0)**	0.00029	-11			HMDB0004949	Ceramides	Lipid
**2S,3R-dihydroxybutyrate**	0.00044	-14	0.054	-10	HMDB0002453	Fatty Acid, Dihydroxy	Lipid
**methyl-4-hydroxybenzoate sulfate**	0.00047	-60			HMDB0041646	Benzoate Metabolism	Xenobiotics
**1-stearoyl-2-arachidonoyl-GPE (18:0/20:4)**	0.00049	-13			HMDB0009003	Phosphatidylethanolamine (PE)	Lipid
**N-acetylarginine**	0.00051	-17			HMDB0004620	Urea cycle; Arginine and Proline Metabolism	Amino Acid

**1-palmitoyl-2-arachidonoyl-GPE (16:0/20:4)***	0.00057	-16			HMDB0008937	Phosphatidylethanolamine (PE)	Lipid
**decadienedioic acid (C10:2-DC)****	0.00083	-41			HMDB0242172	Fatty Acid, Dicarboxylate	Lipid
**1-oleoyl-2-arachidonoyl-GPE (18:1/20:4)***	0.00096	-17			HMDB0009069	Phosphatidylethanolamine (PE)	Lipid
**3-hydroxyoctanoylcarnitine (1)**	0.0010	-23	0.091	-8	HMDB0061634	Fatty Acid Metabolism (Acyl Carnitine, Hydroxy)	Lipid
**urea**	0.0013	-10			HMDB0000294	Urea cycle; Arginine and Proline Metabolism	Amino Acid
**palmitoleoylcarnitine (C16:1)***	0.0015	-12			HMDB0240782	Fatty Acid Metabolism (Acyl Carnitine, Monounsaturated)	Lipid
**tetradecadienedioate (C14:2-DC)***	0.0016	-21				Fatty Acid, Dicarboxylate	Lipid
**N-acetylglucosaminylasparagine**	0.0016	-15			HMDB0000489	Aminosugar Metabolism	Carbohydrate
**1-palmitoyl-2-linoleoyl-GPE (16:0/18:2)**	0.0017	-20			HMDB0008928	Phosphatidylethanolamine (PE)	Lipid
**5-(galactosylhydroxy)-L-lysine**	0.0033	-39	0.032	-19	HMDB0242167	Lysine Metabolism	Amino Acid
**1-stearoyl-2-docosahexaenoyl-GPE (18:0/22:6)***	0.0042	-17			HMDB0009012	Phosphatidylethanolamine (PE)	Lipid
**3-indoxyl sulfate**	0.0051	-20			HMDB0000682	Tryptophan Metabolism	Amino Acid
**bilirubin degradation product, C17H18N2O4 (1)****	0.0056	-24				Partially Characterized Molecules	Partially Characterized Molecules
**2-hydroxybutyrate/2-hydroxyisobutyrate**	0.0059	-10			HMDB0000008	Glutathione Metabolism	Amino Acid
**1-stearoyl-2-linoleoyl-GPE (18:0/18:2)***	0.0060	-16			HMDB0008994	Phosphatidylethanolamine (PE)	Lipid
**alpha-tocopherol**	0.0066	-5			HMDB0001893	Tocopherol Metabolism	Cofactors and Vitamins
**1-methylhistidine**	0.0070	-8			HMDB0000001	Histidine Metabolism	Amino Acid
**butyrylcarnitine (C4)**	0.0072	-48			HMDB0002013	Fatty Acid Metabolism (also BCAA Metabolism)	Lipid
**5-dodecenoylcarnitine (C12:1)**	0.0078	-19			HMDB0013326	Fatty Acid Metabolism (Acyl Carnitine, Monounsaturated)	Lipid
**pregnenetriol sulfate***	0.0079	-19				Pregnenolone Steroids	Lipid
**citrulline**	0.0081	-6			HMDB0000904	Urea cycle; Arginine and Proline Metabolism	Amino Acid
1-palmitoyl-2-oleoyl-GPE (16:0/18:1)	0.0084	-14			HMDB0008927	Phosphatidylethanolamine (PE)	Lipid
3-hydroxyoleoylcarnitine	0.0090	-20				Fatty Acid Metabolism (Acyl Carnitine, Hydroxy)	Lipid
6-hydroxyindole sulfate	0.0093	-23			HMDB0240651	Chemical	Xenobiotics

oleoyl-arachidonoyl-glycerol (18:1/20:4) [2]*	0.0098	-13			HMDB0007228	Diacylglycerol	Lipid
N-acetylphenylalanine	0.010	-15			HMDB0000512	Phenylalanine Metabolism	Amino Acid
myristoylcarnitine (C14)	0.011	-12	0.065	-6	HMDB0254979	Fatty Acid Metabolism (Acyl Carnitine, Long Chain Saturated)	Lipid
gamma-glutamylcitrulline*	0.011	-12				Gamma-glutamyl Amino Acid	Peptide
bilirubin (E,Z or Z,E)*	0.012	-19			HMDB0000488	Hemoglobin and Porphyrin Metabolism	Cofactors and Vitamins
1-stearoyl-GPE (18:0)	0.013	-6			HMDB0011130	Lysophospholipid	Lipid
glycoursodeoxycholate	0.013	-36			HMDB0000708	Secondary Bile Acid Metabolism	Lipid
PE(P-18:0/18:2)*	0.013	-11			HMDB0011376	Plasmalogen	Lipid
glycosyl-N-behenoyl-sphingadienine (d18:2/22:0)*	0.014	-8				Hexosylceramides (HCER)	Lipid
acetylcarnitine (C2)	0.014	-4			HMDB0000201	Fatty Acid Metabolism (Acyl Carnitine, Short Chain)	Lipid
3-methyl-2-oxobutyrate	0.015	-6			HMDB0000019	Leucine, Isoleucine and Valine Metabolism	Amino Acid
bilirubin (Z,Z)	0.016	-13			HMDB0000054	Hemoglobin and Porphyrin Metabolism	Cofactors and Vitamins
N,N-dimethyl-pro-pro	0.016	-5				Modified Peptides	Peptide
N-acetyltyrosine	0.016	-12			HMDB0000866	Tyrosine Metabolism	Amino Acid
4-methyl-2-oxopentanoate	0.017	-8			HMDB0000695	Leucine, Isoleucine and Valine Metabolism	Amino Acid
oleoylcarnitine (C18:1)	0.017	-7			HMDB0005065	Fatty Acid Metabolism (Acyl Carnitine, Monounsaturated)	Lipid
imidazole lactate	0.018	-11			HMDB0002320	Histidine Metabolism	Amino Acid
N-acetylcitrulline	0.019	-20			HMDB0000856	Urea cycle; Arginine and Proline Metabolism	Amino Acid
N-stearoyl-sphingadienine (d18:2/18:0)*	0.021	-16				Ceramides	Lipid
ethylmalonate	0.021	-15			HMDB0000622	Leucine, Isoleucine and Valine Metabolism	Amino Acid
1-stearoyl-2-linoleoyl-GPC (18:0/18:2)*	0.021	-3			HMDB0008039	Phosphatidylcholine (PC)	Lipid
pregnenediol sulfate (C21H34O5S)*	0.021	-18				Pregnenolone Steroids	Lipid
palmitoylcarnitine (C16)	0.021	-7			HMDB0000222	Fatty Acid Metabolism (Acyl Carnitine, Long Chain Saturated)	Lipid
propionylcarnitine (C3)	0.024	-7			HMDB0000824	Fatty Acid Metabolism (also BCAA Metabolism)	Lipid

ximenoylcarnitine (C26:1)*	0.024	-6	0.051	-7		Fatty Acid Metabolism (Acyl Carnitine, Monounsaturated)	Lipid
S-methylcysteine	0.025	-8			HMDB0002108	Methionine, Cysteine, SAM and Taurine Metabolism	Amino Acid
N-acetyl-1-methylhistidine*	0.025	-16			HMDB0240340	Histidine Metabolism	Amino Acid
1-palmitoyl-2-linoleoyl-GPC (16:0/18:2)	0.029	-2			HMDB0007973	Phosphatidylcholine (PC)	Lipid
1-palmitoyl-2-oleoyl-GPC (16:0/18:1)	0.029	-3			HMDB0007972	Phosphatidylcholine (PC)	Lipid
3-methyl-2-oxovalerate	0.030	-7			HMDB0000491	Leucine, Isoleucine and Valine Metabolism	Amino Acid
cortisone	0.031	-10			HMDB0002802	Corticosteroids	Lipid
1-palmitoyl-GPE (16:0)	0.032	-7			HMDB0011503	Lysophospholipid	Lipid
phenylalanine	0.033	-3			HMDB0000159	Phenylalanine Metabolism	Amino Acid
myristoleoylcarnitine (C14:1)*	0.034	-15			HMDB0240588	Fatty Acid Metabolism (Acyl Carnitine, Monounsaturated)	Lipid
branched-chain, straight-chain, or cyclopropyl 12:1 fatty acid*	0.035	-13				Partially Characterized Molecules	Partially Characterized Molecules
N6,N6,N6-trimethyllysine	0.037	-6			HMDB0001325	Lysine Metabolism	Amino Acid
3-hydroxyoctanoylcarnitine (2)	0.037	-13			HMDB0061634	Fatty Acid Metabolism (Acyl Carnitine, Hydroxy)	Lipid
oleoyl-linoleoyl-glycerol (18:1/18:2) [2]	0.038	-8			HMDB0007219	Diacylglycerol	Lipid
cortisol	0.042	-10			HMDB0000063	Corticosteroids	Lipid
1-oleoyl-2-linoleoyl-GPE (18:1/18:2)*	0.043	-18			HMDB0009060	Phosphatidylethanolamine (PE)	Lipid
palmitoyl dihydrosphingomyelin (d18:0/16:0)*	0.043	-5			HMDB0010168	Dihydrosphingomyelins	Lipid
N2-acetyl,N6-methyllysine	0.043	-20			HMDB0242186	Lysine Metabolism	Amino Acid
N-acetylglutamine	0.047	-10			HMDB0006029	Glutamate Metabolism	Amino Acid
bilirubin degradation product, C17H20N2O5 (2)**	0.048	-23				Partially Characterized Molecules	Partially Characterized Molecules
beta-hydroxyisovaleroylcarnitine	0.049	-8	0.097	-5		Leucine, Isoleucine and Valine Metabolism	Amino Acid
deoxycarnitine	0.049	-4			HMDB0001161	Carnitine Metabolism	Lipid

Significance was calculated by multivariate significance (two-sided, P-value w presented) for NSHDS and by paired sample *t*-test (two-sided) for EPIC. Metabolites in bold were also significant after using Benjamini-Hochberg procedure for controlling false discovery rate of 0.2. Identities of metabolites denoted with * or ** have not been confirmed using standards. The identities were still assigned with confidence for metabolites denoted with * and with reasonable confidence for metabolites denoted with **. The values for significant metabolites (P<0.05) and metabolites with P<0.10 in EPIC are given.
[a]4-hydroxyglutamate was lower in cases compared to controls in EPIC.

Supplemental Table 3. Significant metabolites in case-control pairs within two years to diagnosis in NSHDS (n=28 pairs).

Metabolite	P-value	Mean % difference	HMDB ID	Sub-pathway	Super pathway
Higher in cases					
arabitol/xylitol	0.0013	19	HMDB0002917	Pentose Metabolism	Carbohydrate
**erythritol**	0.0025	40	HMDB0002994	Food Component/Plant	Xenobiotics
myo-inositol	0.0028	15	HMDB0000211	Inositol Metabolism	Lipid
**N-acetylvaline**	0.0044	14	HMDB0011757	Leucine, Isoleucine and Valine Metabolism	Amino Acid
2,3-dihydroxyisovalerate	0.0085	82	HMDB0012141	Food Component/Plant	Xenobiotics
**alpha-ketoglutarate**	0.0089	19	HMDB0000208	TCA Cycle	Energy
**erythronate***	0.0098	13	HMDB0000613	Aminosugar Metabolism	Carbohydrate
1-palmitoyl-GPE (16:0)	0.011	12	HMDB0011503	Lysophospholipid	Lipid
**N-lactoyl phenylalanine**	0.015	23	HMDB0062175	Phenylalanine Metabolism	Amino Acid
**N-acetylserine**	0.016	13	HMDB0002931	Glycine, Serine and Threonine Metabolism	Amino Acid
**bilirubin (Z,Z)**	0.018	26	HMDB0000054	Hemoglobin and Porphyrin Metabolism	Cofactors and Vitamins
2-pyrrolidinone	0.021	19	HMDB0002039	Glutamate Metabolism	Amino Acid
pentose acid*	0.024	45	HMDB0000892	Partially Characterized Molecules	Partially Characterized Molecules
**biliverdin**	0.025	25	HMDB0001008	Hemoglobin and Porphyrin Metabolism	Cofactors and Vitamins
N-lactoyl isoleucine	0.027	18	HMDB0062176	Leucine, Isoleucine and Valine Metabolism	Amino Acid
4-hydroxy-2-oxoglutaric acid	0.028	54	HMDB0002070	Fatty Acid, Dicarboxylate	Lipid
cystathionine	0.029	25	HMDB0000099	Methionine, Cysteine, SAM and Taurine Metabolism	Amino Acid
**N-acetylisoleucine**	0.031	14	HMDB0061684	Leucine, Isoleucine and Valine Metabolism	Amino Acid
vanillylmandelate (VMA)	0.031	17	HMDB0000291	Tyrosine Metabolism	Amino Acid
**N-carbamoylvaline**	0.032	28		Leucine, Isoleucine and Valine Metabolism	Amino Acid
**ximenoylcarnitine (C26:1)***	0.034	15		Fatty Acid Metabolism (Acyl Carnitine, Monounsaturated)	Lipid
1-palmitoyl-2-stearoyl-GPC (16:0/18:0)	0.034	8	HMDB0007970	Phosphatidylcholine (PC)	Lipid
suberoylcarnitine (C8-DC)	0.036	77	HMDB0240724	Fatty Acid Metabolism (Acyl Carnitine, Dicarboxylate)	Lipid
retinol (Vitamin A)	0.036	12	HMDB0000305	Vitamin A Metabolism	Cofactors and Vitamins
1-stearoyl-GPE (18:0)	0.037	7	HMDB0011130	Lysophospholipid	Lipid

1-palmitoleoyl-GPC (16:1)*	0.04	13	HMDB0010383	Lysophospholipid	Lipid
**5-methylthioadenosine (MTA)**	0.042	11	HMDB0001173	Polyamine Metabolism	Amino Acid
**N-lactoyl valine**	0.042	18	HMDB0062181	Leucine, Isoleucine and Valine Metabolism	Amino Acid
**2,3-dihydroxy-5-methylthio-4-pentenoate (DMTPA)***	0.042	9	HMDB0240388	Methionine, Cysteine, SAM and Taurine Metabolism	Amino Acid
**isocitric lactone**	0.043	23		TCA Cycle	Energy
**N-acetylalanine**	0.044	8	HMDB0000766	Alanine and Aspartate Metabolism	Amino Acid
bilirubin degradation product, C16H18N2O5 (3)**	0.049	27		Partially Characterized Molecules	Partially Characterized Molecules
2,3-dihydroxy-2-methylbutyrate	0.049	29	HMDB0029576	Leucine, Isoleucine and Valine Metabolism	Amino Acid
Lower in cases					
arachidate (20:0)	0.0074	-18	HMDB0002212	Long Chain Saturated Fatty Acid	Lipid
cis-4-decenoylcarnitine (C10:1)	0.034	-32	HMDB0013205	Fatty Acid Metabolism (Acyl Carnitine, Monounsaturated)	Lipid
cis-4-decenoate (10:1n6)*	0.035	-34	HMDB0004980	Medium Chain Fatty Acid	Lipid
nonadecanoate (19:0)	0.037	-17	HMDB0000772	Long Chain Saturated Fatty Acid	Lipid
**propyl 4-hydroxybenzoate sulfate**	0.041	-237	HMDB0135261	Benzoate Metabolism	Xenobiotics
linoleate (18:2n6)	0.043	-27	HMDB0000673	Long Chain Polyunsaturated Fatty Acid (n3 and n6)	Lipid
sphingomyelin (d18:1/20:1, d18:2/20:0)*	0.047	-14		Sphingomyelins	Lipid
linolenate [alpha or gamma; (18:3n3 or 6)]	0.047	-34	HMDB0001388	Long Chain Polyunsaturated Fatty Acid (n3 and n6)	Lipid
stearate (18:0)	0.048	-22	HMDB0000827	Long Chain Saturated Fatty Acid	Lipid
sphingomyelin (d18:1/25:0, d19:0/24:1, d20:1/23:0, d19:1/24:0)*	0.049	-16		Sphingomyelins	Lipid

Significance was calculated by paired sample *t*-test (two-sided). Metabolites in bold were also significant within eight years to diagnosis within NSHDS. Identities of metabolites denoted with * or ** have not been confirmed using standards. The identities were still assigned with confidence for metabolites denoted with * and with reasonable confidence for metabolites denoted with **.

Supplemental Table 4. Significant metabolites in case-control pairs within two years to diagnosis in EPIC (n=28 pairs).

Metabolite	P-value	Mean % difference	HMDB ID	Sub-pathway	Super Pathway
Higher in cases					
3-phenylpropionate (hydrocinnamate)	0.0083	128	HMDB0000764	Benzoate Metabolism	Xenobiotics
**isobutyrylcarnitine (C4)**	0.011	34	HMDB0000736	Leucine, Isoleucine and Valine Metabolism	Amino Acid
cinnamoylglycine	0.014	96	HMDB0011621	Food Component/Plant	Xenobiotics
lithocholate sulfate (1)	0.029	81		Secondary Bile Acid Metabolism	Lipid
benzoate	0.031	12	HMDB0001870	Benzoate Metabolism	Xenobiotics
indolin-2-one	0.033	45		Food Component/Plant	Xenobiotics
2-aminophenol sulfate	0.038	42	HMDB0061116	Food Component/Plant	Xenobiotics
3-sulfo-L-alanine	0.046	25	HMDB0002757	Methionine, Cysteine, SAM and Taurine Metabolism	Amino Acid
androstenediol (3alpha, 17alpha) monosulfate (3)	0.048	19		Androgenic Steroids	Lipid
epiandrosterone sulfate	0.049	32	HMDB0062657	Androgenic Steroids	Lipid
Lower in cases					
1-palmitoyl-2-linoleoyl-GPI (16:0/18:2)	0.029	-42	HMDB0009784	Phosphatidylinositol (PI)	Lipid
oleoyl-linoleoyl-glycerol (18:1/18:2) [1]	0.033	-61	HMDB0007219	Diacylglycerol	Lipid
**decadienedioic acid (C10:2-DC)****[, A]	0.043[a]	-88	HMDB0242172	Fatty Acid, Dicarboxylate	Lipid
**PE(P-18:0/18:2)***	0.044	-15	HMDB0011376	Plasmalogen	Lipid
perfluorooctanoate (PFOA)	0.048	-17	HMDB0059587	Chemical	Xenobiotics

Significance was calculated by paired sample *t*-test (two-sided). Metabolites in bold were also significant within eight years to diagnosis in NSHDS. Identities of metabolites denoted with * or ** have not been confirmed using standards. The identities were still assigned with confidence for metabolites denoted with * and with reasonable confidence for metabolites denoted with **.

[A] Decadienedioic acid (C10:2-DC)** was elevated in cases compared to controls in NSHDS.

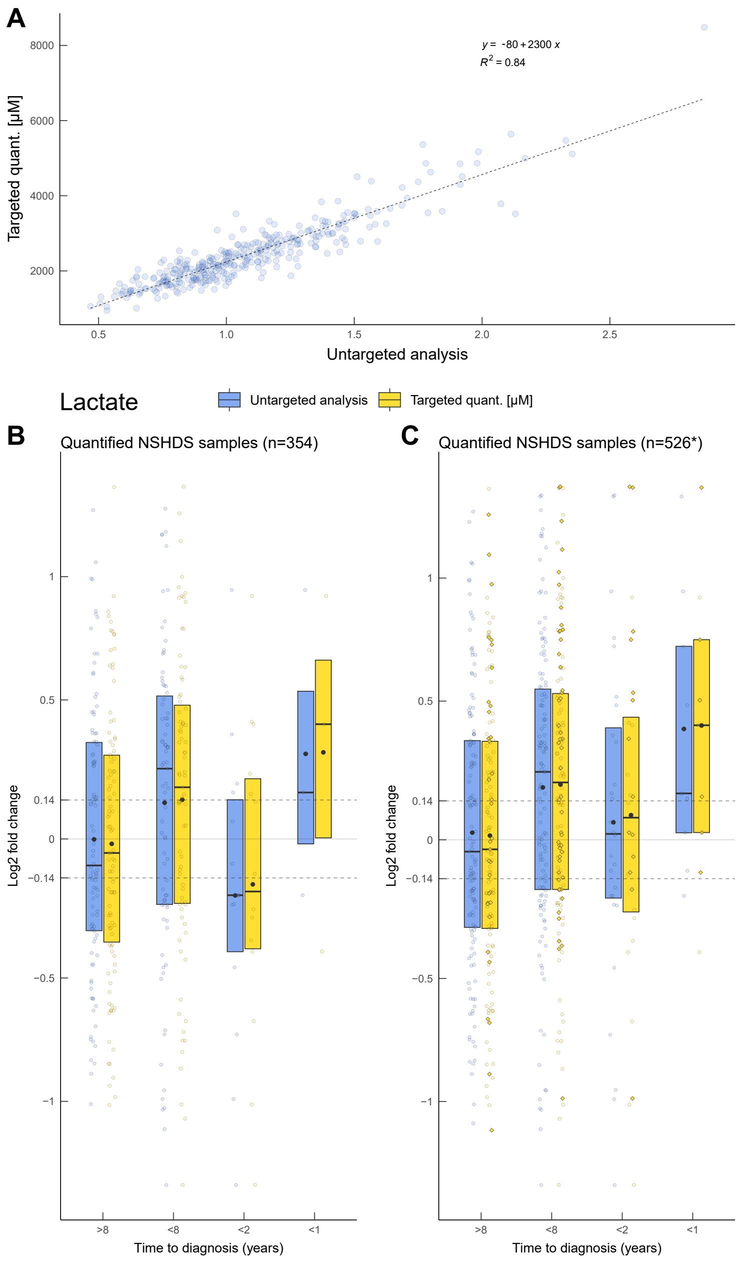

Supplemental Figure 1. Validation of lactate concentrations in plasma by targeted quantification. (**A**) Linear regression plot for lactate levels in 354 NSHDS plasma samples using Metabolon Inc. untargeted global metabolomics analysis and by targeted Biocrates MxP® Quant 500 analysis. (**B**) Boxplot with average (dot) and median (line) fold change of lactate in the NSHDS samples (n=354) measured using Metabolon Inc. untargeted analysis (blue) and by targeted Biocrates MxP® Quant 500 (yellow). Case-control pairs are subgrouped according to time to diagnosis (>8 years n=98, <8 years n=79, <2 years n=14, <1 year n=3). Dashed horizontal lines display a 10 % difference. The Y-axis is non-linearly transformed. (**C**) Boxplot as shown in **B**. with all NSHDS samples (n=526) measured using untargeted analysis (blue) and by targeted analysis (yellow), * including predicted case-control fold changes based on extrapolated values from the linear regression shown in **A**. (yellow diamonds, n=86). Case-control pairs are subgrouped according to time to diagnosis (>8 years n=133, <8 years n=130, <2 years n=28, <1 year n=9).

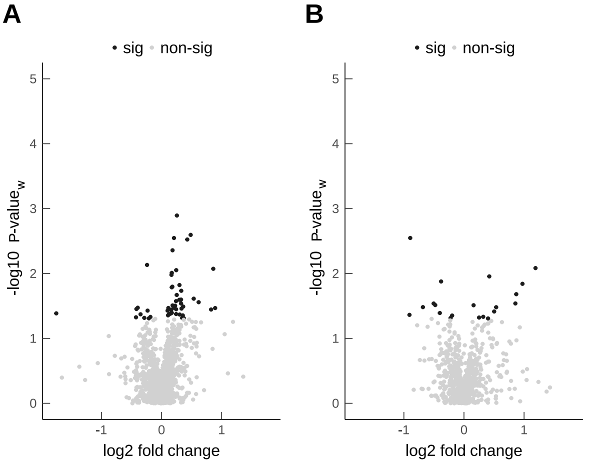

Supplemental Figure 2. Metabolite significance and fold change for case-control pairs within two years to diagnosis. Volcano plots of detected molecular features in (**A**) NSHDS (n=28 pairs) and (**B**) EPIC (n=28 pairs) within two years to diagnosis, with effect sizes and significance levels for each molecular feature as log-ratios. Significance was calculated by multivariate significance (two-sided, P-value$_w$ plotted). Sig = Significant molecular features; non-sig = non-significant molecular features.

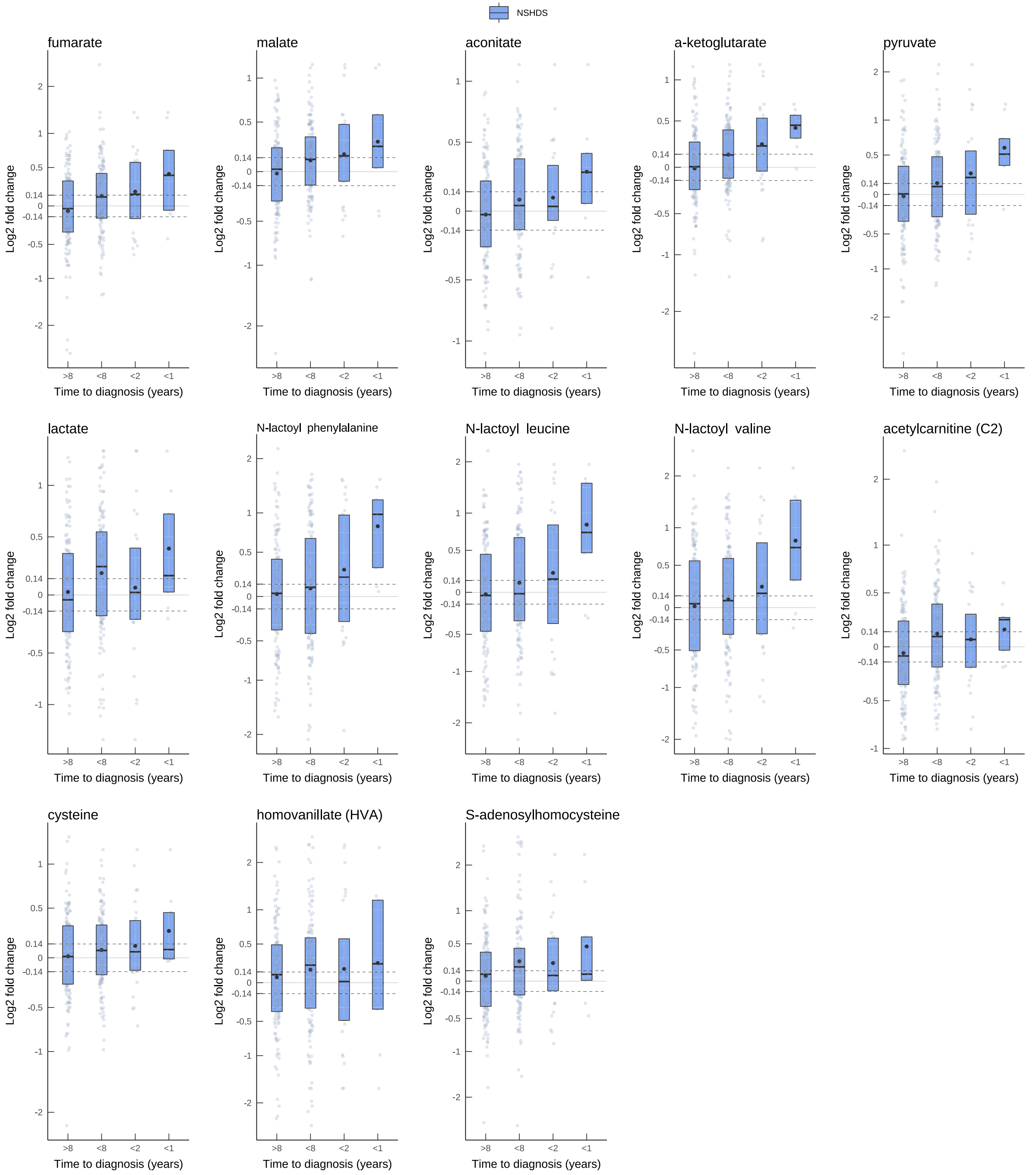

Supplemental Figure 3. Metabolite levels for case-control pairs towards diagnosis, within significant pathways. Boxplots with average (dot) and median (line) fold change for case-control pairs within NSHDS, subgrouped according to time to diagnosis (>8 years: n=133. <8 years: n=130. <2 years: n=28. <1 year: n=9). Dashed horizontal lines display a 10% difference. The y-axis is non-linearly transformed.

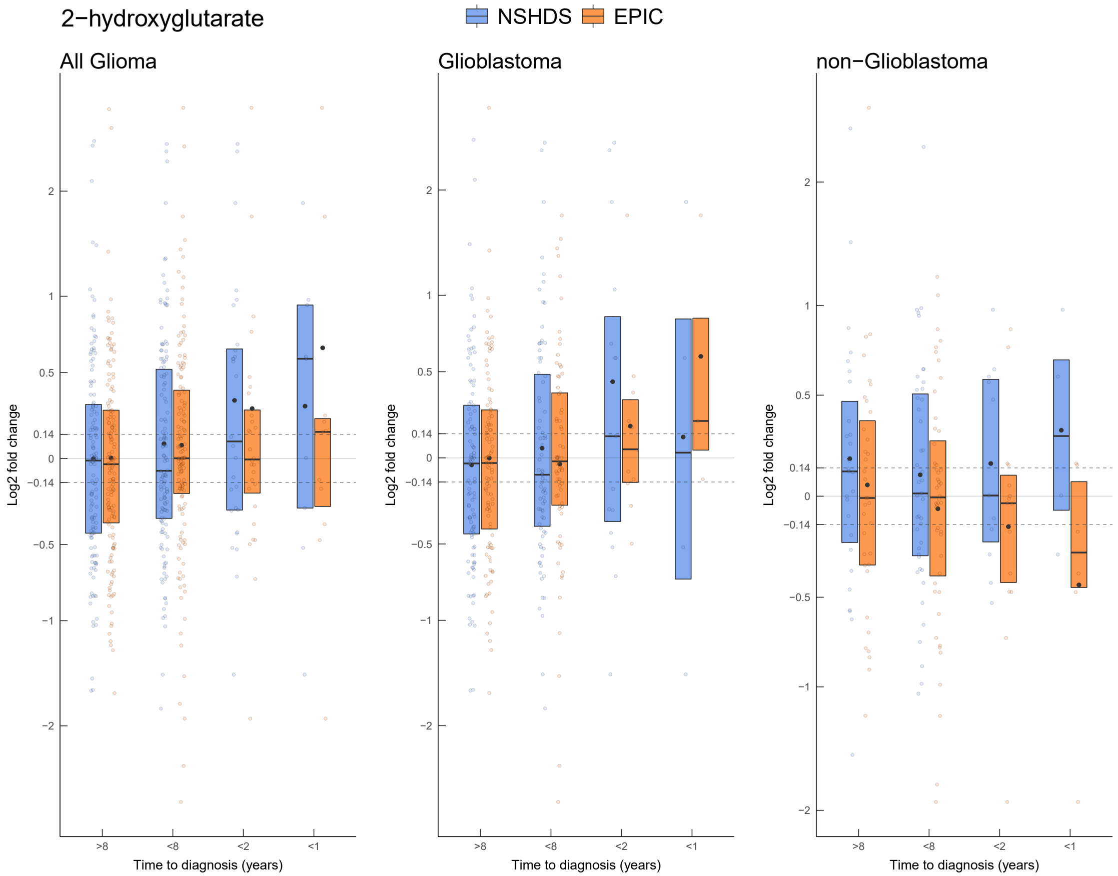

Supplemental Figure 4. 2-hydroxyglutarate levels for case-control pairs towards diagnosis. Boxplots with average (dot) and median (line) fold change of 2-hydroxyglutarate in case-control pairs for NSHDS (blue) and EPIC (orange) samples, subgrouped according diagnosis and to time to diagnosis (all glioma: >8 years: NSHDS, n=133 and EPIC, n=148. <8 years: NSHDS, n=130 and EPIC, n=139. <2 years: NSHDS, n=28 and EPIC, n=28. <1 year: NSHDS, n=9 and EPIC, n=11. Glioblastoma: >8 years: NSHDS, n=105 and EPIC, n=100. <8 years: NSHDS, n=79 and EPIC, n=70. <2 years: NSHDS, n=15 and EPIC, n=10. <1 year: NSHDS, n=4 and EPIC, n=3. non-glioblastoma: >8 years: NSHDS, n=27 and EPIC, n=35. <8 years: NSHDS, n=43 and EPIC, n=48. <2 years: NSHDS, n=11 and EPIC, n=15. <1 year: NSHDS, n=4 and EPIC, n=6). Dashed horizontal lines display a 10 % difference. The Y-axis is non-linearly transformed.
